# Post-transcriptional control of a stemness signature by RNA-binding protein MEX3A regulates murine adult neurogenesis

**DOI:** 10.1038/s41467-023-36054-6

**Published:** 2023-01-23

**Authors:** Ana Domingo-Muelas, Pere Duart-Abadia, Jose Manuel Morante-Redolat, Antonio Jordán-Pla, Germán Belenguer, Jaime Fabra-Beser, Lucía Paniagua-Herranz, Ana Pérez-Villalba, Adrián Álvarez-Varela, Francisco M. Barriga, Cristina Gil-Sanz, Felipe Ortega, Eduard Batlle, Isabel Fariñas

**Affiliations:** 1 Departamento de Biología Celular, Biología Funcional y Antropología Física, Universidad de Valencia, Valencia, Spain; 2 Instituto de Biotecnología y Biomedicina (BIOTECMED), Universidad de Valencia, Valencia, Spain; 3 Centro de Investigación Biomédica en Red sobre Enfermedades Neurodegenerativas (CIBERNED), Valencia, Spain; 4 Departamento de Bioquímica y Biología Molecular, Universidad Complutense de Madrid (UCM), Madrid, Spain; 5 Instituto Universitario de Investigación en Neuroquímica (IUIN), Madrid, Spain; 6 Instituto de Investigación Sanitaria San Carlos (IdISSC), Madrid, Spain; 7 Institute for Research in Biomedicine (IRB Barcelona), Barcelona, Spain; 8 Centro de Investigación Biomédica en Red de Cáncer (CIBERONC), Barcelona, Spain; 9 ICREA, Barcelona, Spain

**Keywords:** Adult neurogenesis, Molecular neuroscience

## Abstract

Neural stem cells (NSCs) in the adult murine subependymal zone balance their self-renewal capacity and glial identity with the potential to generate neurons during the lifetime. Adult NSCs exhibit lineage priming via pro-neurogenic fate determinants. However, the protein levels of the neural fate determinants are not sufficient to drive direct differentiation of adult NSCs, which raises the question of how cells along the neurogenic lineage avoid different conflicting fate choices, such as self-renewal and differentiation. Here, we identify RNA-binding protein MEX3A as a post-transcriptional regulator of a set of stemness associated transcripts at critical transitions in the subependymal neurogenic lineage. MEX3A regulates a quiescence-related RNA signature in activated NSCs that is needed for their return to quiescence, playing a role in the long-term maintenance of the NSC pool. Furthermore, it is required for the repression of the same program at the onset of neuronal differentiation. Our data indicate that MEX3A is a pivotal regulator of adult murine neurogenesis acting as a translational remodeller.

## Abstract (web summary)

Here the authors identify RNA-binding protein MEX3A as a post-transcriptional regulator of quiescence and differentiation signatures at critical transitions along the murine subependymal neurogenic lineage

## Introduction

**Fig. 1:**
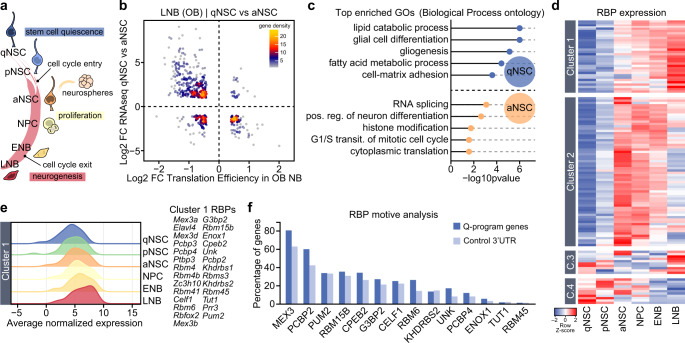
Post-transcriptional control of a NSC stemness/gliogenic signature during neural lineage differentiation. **a** Schematic drawing representing the cell populations in the adult SEZ neurogenic lineage (qNSC: quiescent neural stem cells; pNSC: primed NSC; aNSC: activated NSC; NPC: neural progenitor cell; ENB: early neuroblast; LNB: late neuroblast). Adult NSC can be cultured and expanded in vitro as clonal aggregates, neurospheres. Cell cycle entry and exit points along the lineage are highlighted. **b** Scatterplot comparing differential expression (DE) data from isolated qNSC vs. aNSC populations^12^ with translation efficiency data from OB NBs^17^. **c** Gene Ontology (GO) enrichment analysis of genes that define the qNSC and aNSC signature and are subjected to translational repression during lineage differentiation. **d** Heatmap showing the expression pattern of different families of RNA-binding proteins (RBPs) in isolated SEZ populations^12^. **e** Ridgeplot showing average expression of Cluster 1 RBPs in SEZ populations. **f** Cluster 1 RBP motive analysis in qNSC-signature genes that are translationally repressed during neuroblast differentiation. Graph shows the percentage of 3’UTR sequences that include the RBP motive compared to a set control 3’UTR.

The adult mammalian brain is a highly pro-gliogenic environment. While oligodendrocyte generation by NG2^+^ progenitor cells occurs widespread, postnatal neurogenesis is remarkably restricted to two specific locations^1^. Neural stem cells (NSCs) present in the neurogenic niches of the subependymal zone (SEZ) and subgranular zone (SGZ) are also of glial nature but generate neurons, for the olfactory bulb (OB) and the dentate gyrus respectively. Astrocyte-like subependymal NSCs include deeply quiescent (qNSCs) and shallow quiescent/primed NSCs (pNSCs) which eventually become activated NSCs (aNSCs) that generate transit-amplifying neural progenitor cells (NPCs). These progenitors divide a few times before they turn into proliferating early neuroblasts (ENBs) that migrate anteriorly, via the rostral migratory stream (RMS), and eventually exit the cell cycle as late NBs (LNBs) to terminally differentiate into neurons in the OB^2^ (Fig. 1a). At the origin of this orderly program of cell commitment, adult NSCs exhibit lineage priming, with levels of pro-neurogenic fate determinants that are higher than in other brain glial cells^3^. These levels, however, are not enough to drive the direct differentiation of adult NSCs into neurons and their descendants need to gradually acquire a complete and effective set of neural fate determinants for terminal differentiation^4,5^. This raises the intriguing question of how cells along the neurogenic lineage avoid the critical challenge posed by genes that encode proteins with conflicting function, i.e., self-renew or differentiate^6^. A few repressive transcriptional mechanisms have been proposed to counteract lineage priming as a way to preserve stemness^7–11^. However, little attention has been devoted to post-transcriptional mechanisms.

Gene regulation analysis in cell fate studies has traditionally been centered on transcription. In the SEZ, next generation sequencing has accurately revealed the transcriptomes associated to different cellular states and/or identities^3,12–15^ and yet, they are not fully coherent with recently characterized global proteomes^16^. More specifically, a recent report experimentally analyzed global concordance between transcription and translation in the neurogenic lineage^17^. The authors crossed R26R-Tomato mice carrying either a *Tlx-Cre* (NSCs) or a *Doublecortin* (*Dcx)-Cre* (NBs) recombinase transgene with RiboTag mice, in which the endogenous coding sequence for the ribosomal large subunit protein RPL22 is replaced by a Cre-recombinase dependent HA-tagged variant^18^. Deep sequencing of the total RNA (transcriptome) and of the HA-immunoprecipitated ribosome-bound RNA (translatome) in TLX^+^ NSCs and in DCX^+^ NBs revealed an uncoupling between transcription and translation in the latter^17^. The data suggested an adaptive translatome remodeling at the NB stage. However, regulators of this process remain elusive.

Global adaptations in protein output are emerging as a fundamental step in cellular adaptation to fast evolving situations, i.e. in response to cellular stress^6,19–21^. Classic regulators of mRNA stability and translation efficiency involve non-coding RNAs and RNA-binding proteins (RBPs), which can act as translatome remodelers^19,22,23^. Among all protein-coding genes in humans 7.5% are RBPs, amounting to more than 1500 different ones. An RBP can regulate the coordinated translation of a pool of transcripts coding for proteins that together participate in a cellular process, but specific effects and “RNA regulons” await characterization in most cases^19,24–26^. In the present work, we in silico identify MEX3A as the RBP with more binding sequences in transcripts that are translationally repressed at the onset of differentiation in the SEZ lineage. MEX3A target transcripts identified by RNA immunoprecipitation followed by deep sequencing (RIP-seq) reveal that MEX3A post-transcriptionally regulates genes associated with quiescence and glial identity in a dose-dependent manner. Low levels of MEX3A in recently activated NSCs sustain their capacity to return to quiescence whereas high levels at the end of the lineage repress a stemness signature, allowing NBs to timely exit the cell cycle for differentiation. With a role in two important cell transitions in the neurogenic niche, MEX3A is essential for the lifelong maintenance of neurogenesis.

## Results

### An unbiased in silico search for functionally relevant RBPs in adult neurogenesis identifies MEX3A

In order to identify RBPs that might be regulating cell lineage progression during SEZ neurogenesis, we first searched our recently published transcriptomic data sets on quiescent dormant (q) NSCs, shallow quiescent or primed (p) NSCs, and activated (a) NSCs^12^ for genes exhibiting uncoupling between their ribosome-bound and unbound mRNAs in the Baser et al. (2019) study. The comparison revealed that the set of transcripts translationally repressed in LNB include 84 of the genes differentially upregulated in aNSCs and a larger set of mRNAs (253 genes) that are characteristically expressed by both qNSCs and pNSCs (Fig. 1b). Gene Ontology (GO) analysis indicated that the repressed mRNAs that characterized aNSCs were related to splicing, translation and proliferation. Moreover, translationally-repressed mRNAs of the quiescent signature corresponded to genes involved in gliogenesis, glial identity and lipid metabolism (Fig. 1c). The data indicated that a number of translationally-repressed genes at the onset of neuronal differentiation are part of a NSC quiescent/gliogenic signature maintained during lineage progression in the subependymal neurogenic niche.

Next, we decided to search in our expression data sets for RBPs whose expression was low in quiescent NSCs but increased along the lineage to achieve high levels in LNB and found that, out of 116 RBPs with differential expression in our populations, 23% of them exhibited the proposed expression pattern (cluster 1 in Fig. 1d, e). These RBPs were highly enriched in K Homology (KH) and RNA Recognition Motif (RMM) domains and functionally related to processes like translation (GO:0006417, *p* = 4.34e-8), splicing (GO:0048024, *p* = 1.14e-12) and post-transcriptional regulation of gene expression (GO:0010608, *p* = 5.05e-10). A search for consensus sequences for these 27 RBPs in the 3´UTR of those quiescence-related mRNAs whose translation was repressed in LNBs indicated that the most represented one was that for proteins of the MEX-3 family (Fig. 1f).

**Fig. 2:**
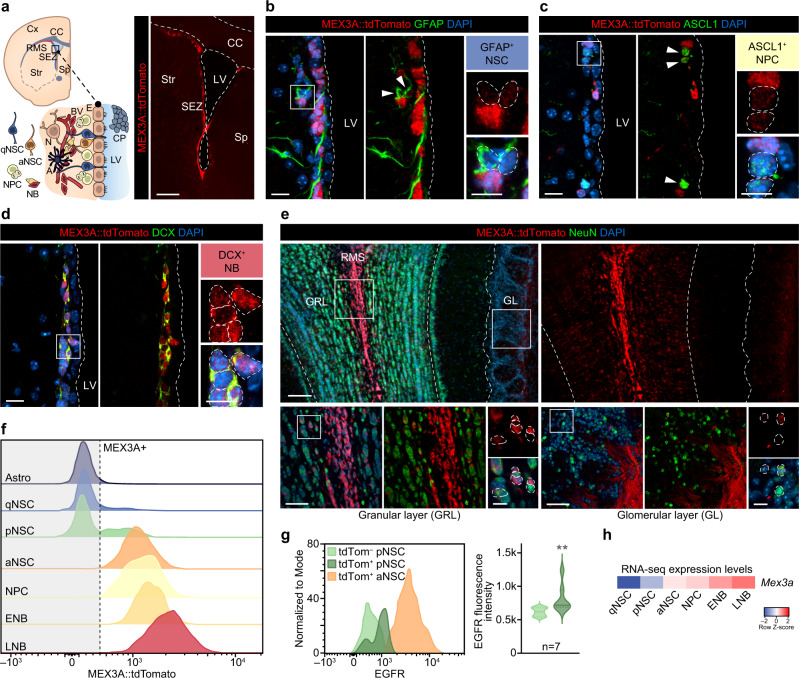
RNA-binding protein MEX3A as a potential regulator at the onset of SEZ lineage activation. **a** Schematic drawing of a coronal brain section displaying the SEZ niche cytoarchitecture (left panel). Immunohistochemistry showing expression of MEX3A::tdTomato (red) in a panoramic image of the adult *Mex3a*^*+/KI*^ SEZ (right panel). **b** Immunohistochemistry showing expression of MEX3A::tdTomato (red) in GFAP^+^ (green) NSCs. **c** Immunohistochemistry showing expression of MEX3A::tdTomato (red) in ASCL1^+^ (green) NPCs. **d** Immunohistochemistry for MEX3A::tdTomato (red) in DCX^+^ (green) NBs. **e** Panoramic view of a coronal immunostained section of the OB showing expression of MEX3A::tdTomato (red) and NeuN (green, mature neurons) (top panel). High-magnification images of MEX3A::tdTomato expression (red) among NeuN^+^ neurons (green) in the granular (GRL) and the glomerular (GL) layers (bottom panels). **f** Representative FACS histograms showing MEX3A::tdTomato expression among SEZ populations. **g** Representative FACS histogram showing EGFR levels in both tdTomato^–^ and tdTomato^+^ pNSCs and in tdTomato^+^ aNSCs (left panel). Quantification of the EGFR median fluorescence intensity in tdTomato^–^ and tdTomato^+^ pNSCs (*p*-value=0.002, *n* = 7 mice, by two-tailed Mann-Whitney U) (right panel). **h** Heatmap showing *Mex3a* RNA-seq expression levels in sorted populations^12^. Arrowheads indicate positive cells. White boxes localize high-magnification inserts. Dashed lines mark the lateral ventricle (LV) and contour the nuclei of positive cells. SEZ: subependymal zone; RMS: rostral migratory stream; CC: corpus callosum; LV: lateral ventricle; Cx: cortex; Str: striatum; Sp: septum; qNSC: quiescent neural stem cell; aNSC: activated NSC; NPC: neural progenitor cell; NB: neuroblast; E: ependymal cell; N: neuron; A: astrocyte; BV: blood vessel; CP: choroid plexus; GRL granular layer; GL: glomerular layer. Source data are provided as a Source Data file. Scale bars: **a**, 300 μm; **b**, **c** and d, 15 μm; **e**, 100 μm and 50 μm; inserts, 10 μm.

MEX-3 (mex: muscle excess), a KH domain RBP, was initially reported to prevent the translation of pro-differentiation genes by retaining their mRNAs in P granules during blastomere asymmetric division in *C. elegans*^27–34^. Among the four family members (MEX3A, B, C, D) in mammals (see expression in our system, Supplementary Fig. 1a, b), only MEX3A has been associated to certain types of cancer^35–38^ and to be highly expressed in a subpopulation of LGR5^+^ (leucine-rich repeat-containing G-protein coupled receptor 5) intestinal stem cells that proliferate slowly and are resistant to chemo- and radiotherapy^39,40^. We, therefore, decided to analyze MEX3A expression by using the same reporter knock-in (KI) mouse strain, carrying a tdTomato/T2A/Cre-ERT2/bGH-polyA construct inserted in frame with the *Mex3a* start codon in exon 1^40^. Immune detection of the tdTomato protein in brain sections of adult *Mex3a*^*+/KI*^ brains showed that MEX3A expression is virtually restricted to neurogenic niches (Fig. 2a and Supplementary Fig. 1c, d). In the SEZ, GFAP^+^ NSCs and ASCL1^+^ NPCs showed faint, albeit specific expression, and DCX^+^ NBs exhibited a very strong signal (Fig. 2b–d), indicating a progressive increase in expression along the neurogenic lineage. A similar pattern was observed in the SGZ (Supplementary Fig. 1d–f). S100β^+^ mature astrocytes and ependymal cells lining the ventricle wall or granular and striatal neurons did not express the reporter (Supplementary Fig. 1f–h), The reporter signal was still strong in the NBs migrating through the RMS, but it became dimmer as they arrived at the OB and migrated radially outwards and NeuN^+^ mature neurons did not show any expression (Fig. 2e and Supplementary Fig. 1i, j). We also analyzed *Mex3a* expression in SEZ cell populations from *Mex3a*^*+/KI*^ reporter mice following a strategy developed in our laboratory to classify all cells of the neurogenic lineage using flow cytometry^12,41^. We found no expression of *Mex3a* in mature astrocytes or in qNSCs (Fig. 2f). The onset of expression correlated with the appearance of the EGFR in a fraction of pNSCs and the level gradually increased along the lineage from aNSCs, to NPCs, to NBs (Fig. 2f, g). The expression pattern was consistent with our transcriptomic data (Fig. 2h) and confirmed that *Mex3a* expression starts in pNSCs, more prone to enter cell cycle, and increases in proliferative populations along the subependymal lineage, becoming maximal at the NB stage.

An in silico search in the promoter of *Mex3a* for binding sites for transcription factors (TFs) revealed 36 putative regulators. Among those that can bind *Mex3a*, TFs in cluster 2 and 3 also exhibited a progressively increased expression along the SEZ lineage (Supplementary Fig. 2a–c). Some of these TFs (Sp8, Smarcc2, Hcfc1) have been shown to play a role in NSC proliferation and differentiation^42–44^, in line with potential functional effects of MEX3A in the SEZ lineage progression.

### Identification of the MEX3A targetome by RIP-seq

**Fig. 3:**
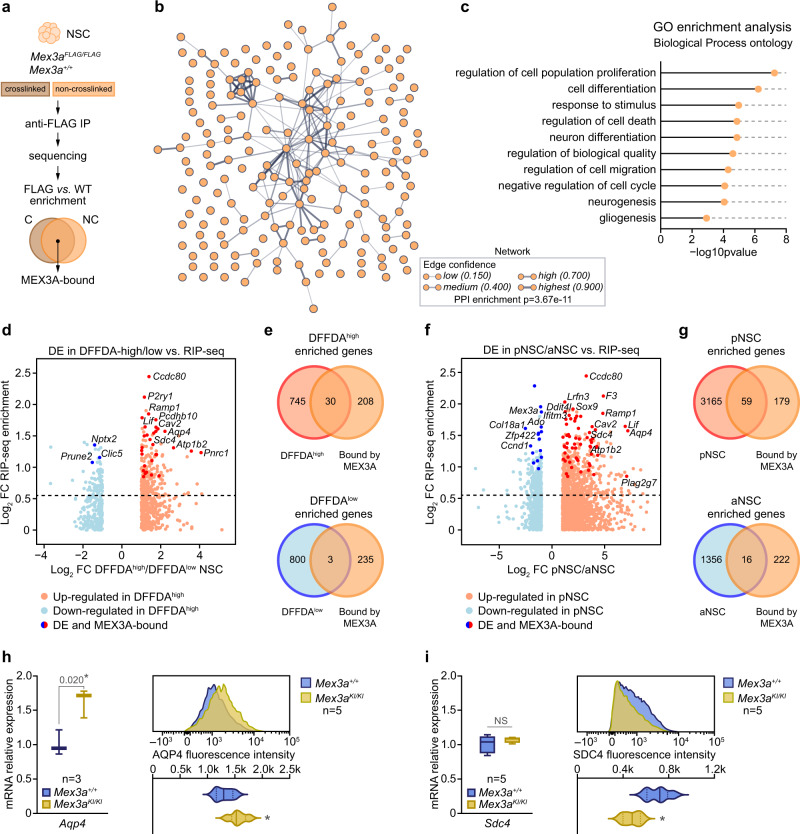
The MEX3A regulon reveals a stemness/gliogenic program associated with neurogenic lineage quality. **a** MEX3A RNA-immunoprecipitation (RIP) diagram. IP: immunoprecipitation; C: crosslinked samples; NC: non-crosslinked samples. **b** Protein-protein interaction network generated from MEX3A-bound coding RNAs (STRING PPI enrichment *p*-value = 3.67 × 10^−11^). Line thickness indicates strength and confidence of the connection (https://string-db.org/). See Supplementary Fig. 3c and Supplementary Data 1 for detailed target lists. **c** Representative Gene Ontology (GO, Biological Process) terms enriched in the subset of MEX3A-bound coding RNAs (see Supplementary Data 2 for a complete GO list). Significance values are reported after FDR correction. **d** Scatterplot comparing differential expression (DE) data from DFFDA^high^ vs. DFFDA^low^ populations^12^ with MEX3A RIP-seq enrichment (FDR < 0.05). Red dots represent up-regulated, and blue dots down-regulated genes in DFFDA^high^ cells. Dark red and blue dots label DE genes also bound by MEX3A. **e** Overlap between MEX3A-bound genes (orange) and genes enriched in DFFDA^high^ (red, top) and DFFDA^low^ (blue, bottom) populations. DFFDA^high^ and MEX3A-bound genes show more overlap than expected (*p*-value = 8.5 × 10^−8^, by hypergeometric test). **f** DE data from isolated pNSC vs. aNSC populations^12^ with MEX3A RIP-seq enrichment data (FDR < 0.05). Red dots represent up-regulated, and blue dots down-regulated genes in pNSCs. Dark red and blue dots label DE genes also bound by MEX3A. **g** Overlap between MEX3A-bound genes (orange) and genes enriched in pNSCs (red, top) and aNSCs (blue, bottom). pNSC and MEX3A-bound genes show more overlap than expected (*p*-value=0.003, by hypergeometric test). Quantification of *Aqp4* (**h**) and *Sdc4* (**i**) gene expression by RT-qPCR in *Mex3a*^*+/+*^ and *Mex3a*^*KI/KI*^ NSCs (left panels) (^*^p-value < 0.05 and NS = 0.317 by unpaired two-tailed Student’s *t*-test). AQP4 (**h**) and SDC4 (**i**) protein levels (median fluorescence intensity) by FACS in wild-type and KI homozygous NSC cultures (right panels) (p-values: AQP4 = 0.032, SDC4 = 0.011, by unpaired two-tailed Student’s *t*-test). Box plots show median± interquartile range and whiskers define minimum to maximum. Exact *p*-values and the number of biologically independent samples used are indicated. Source data are provided as a Source Data file.

Only a few MEX3A target RNAs have been identified in mouse and human tissues^34^. To analyze the MEX3A “RNA regulon” in adult NSCs we generated a FLAG-tagged MEX3A knock-in mouse, which expresses the endogenous MEX3A protein with a 3X-FLAG peptide fused to its C-terminal region, in order to perform RIP-seq experiments with antibodies against the FLAG epitope (Fig. 3a). pNSCs and aNSCs dissociated from the SEZ and seeded under non-adherent conditions in the presence of mitogens epidermal growth factor (EGF; 20 ng/ml) and basic fibroblast growth factor (bFGF; 10 ng/ml) form floating clonal aggregates known as neurospheres that can be expanded for long periods of time (see Methods). In these cultures MEX3A was successfully immunoprecipitated (Supplementary Fig. 3a) in both crosslinked and native conditions and pulled-down RNA was sequenced. Genes with more than 0.58 log_2_ FC (fold-change FC > 1.5 times) and a False Discovery Rate (FDR) adjusted *p*-value < 0.05 were considered as positively enriched in Mex3a-FLAG neurospheres (RIP-enriched) compared to the wild-type background controls (RIP-control). The sets of RIP-enriched genes obtained by both crosslinked and native IP protocols were highly overlapping. We only considered for further analysis those genes that were enriched in both IPs to stringently obtain true MEX3A-bound RNAs. We detected 238 RIP-enriched genes, approximately 82% of which were protein-coding genes and associated in a STRING-inferred functional network (Fig. 3b and Supplementary Fig. 3c; Supplementary Data 1). GO analysis of this MEX3A-dependent RNA regulon revealed that the most relevant GO Biological Process terms were associated with regulation of cell proliferation, cell differentiation and biological quality (Fig. 3c; Supplementary Data 2). Of note, we observed that MEX3A binds to all four *Mex3* family members, including itself, suggesting a fine and controlled feedback regulation in this RBP family (Supplementary Data 1).

In order to determine the position of MEX3A in the transcripts, we compared sequence enrichment of crosslinked *Mex3a*^*FLAG*/*FLAG*^ IP samples *vs. Mex3a*^*+*/*+*^ controls. Protein-coding metagene analysis revealed that MEX3A binds its mRNAs along the gene body, with a clear tendency to accumulate toward the 3’ end (Supplementary Fig. 3d). MEX3A binding was maximally enriched around the last 500 bp up to the pA site (Supplementary Fig. 3e), placing MEX3A preferentially on the 3’UTR of these RNAs. Expectedly, RIP-seq values in MEX3A-bound genes far exceeded that of the average of protein-coding genes (Supplementary Fig. 3d, e). Analysis of the 3’UTRs of the 196 RIP-enriched protein-encoding mRNAs, and of 200 control mRNAs of similar length, for the presence of the MEX-3 recognition element (MRE) consensus sequence in *C. elegans* (DKAGN(0-8)THAT^45^) revealed an statistically significant enrichment in MEX3A-pulled-down RNAs (Supplementary Fig. 3f), validating the specificity of the RIP experiments. Identification of protein-associated complexes by mass spectrometry (LC-MS/MS) after co-immunoprecipitation (Supplementary Fig. 4a) uncovered 22 proteins that were associated to MEX3A and its regulon (Supplementary Fig. 4b). These include: factors involved in transcription and splicing (hnRNPs), factors associated to mRNA decay (UPF1), poly-A binding proteins (PABPC1), ribosomal proteins and translation initiation and elongation factors (RPL30, EIF4A2, EEF2) and other RBPs (ELAVL1, QK1, FUBP3). All of them are related to post-transcriptional regulation and translation. The protein interactome suggests that MEX3A likely associates to different sets of factors to regulate translation and that it does so in combination with other RBPs, picturing a highly complex scenario.

### A MEX3A regulon reveals its involvement in the preservation of a quiescence signature during reversible activation

To search in silico for specific gene expression programs regulated by MEX3A we based our premises on its pattern of expression. The initial activation of *Mex3a* expression in pNSCs (Fig. 2f, g) prompted us to explore the potential existence of a ‘quiescence/stemness program’ that needs to be controlled by MEX3A when NSCs become activated. Because RIP-seq data was obtained from neurospheres, we decided to first compare the RIP-enriched data set with the transcriptomes of neurosphere cells that cycle at different pace. We have recently described that growing neurospheres contain fast-dividing cells and a lower proportion of slowly dividing cells with a primed-like molecular signature that can retain fluorophores like the green fluorescent DFFDA^12^. Single cells loaded with DFFDA dilute the tracer in each division, as the label is passively distributed between sibling cells. Whereas most cells in grown neurospheres have expectedly lost the label (DFFDA^low^), around half of all spheres contain a few cells that still retain a sufficient amount of the fluorophore to be detected as DFFDA^high^, indicating that their initiating cells had divided only a few times before they stopped or slowed down their cycling. Comparison of the subsets of genes that define the DFFDA^high^ (pNSC-like molecular signature) and DFFDA^low^ (aNSC-like molecular profile) populations in vitro^12^ with our MEX3A RIP-seq enrichment data showed that MEX3A preferentially binds RNAs that are up-regulated in the slowly-dividing DFFDA^high^ population (*p* = 8.5e-8) (Fig. 3d, e). We used the same approach with the populations in vivo by using the RNA-seq data obtained from acutely isolated pNSCs and aNSCs^12^. Among the thousands of genes that characterize the ‘pNSC to aNSC’ transition, MEX3A-bound genes showed a significant overlap with the pNSC profile (*p* = 0.003) (Fig. 3f, g), indicating again that MEX3A binds a set of ‘quiescence-associated genes’. The program includes genes that inhibit proliferation in adult neurogenic niches (*Apoe, Cav2, Pla2g7*) or are related to NSC identity (*Aqp4, Plec, Lif, Sox9*)^46–53^ among others. The data indicated that MEX3A becomes expressed and binds a set of quiescence-related genes as NSCs enter activation.

Complete elimination of *Mex3a* results in perinatal lethality (unpublished data). Still, homozygous knock-in mice survive into adulthood, but insertion of the KI reporter cassette in the two alleles results in a decrease of more than 90% in *Mex3a* mRNA and MEX3A protein levels, with no apparent changes in the levels of other family members (Supplementary Fig. 5a–c), providing a way to analyze MEX3A specific functions. To validate the repressive effect of MEX3A on the translation of these quiescence-associated target genes, we selected the *Aquaporin-4* (*Aqp4*) gene since it exhibited the largest compound fold-change in both the ‘pNSC to aNSC’ and ‘DFFDA^high^ to DFFDA^low^’ transitions. MEX3A-deficient NSCs showed increased levels of *Aqp4* mRNA and protein (Fig. 3h). While MEX3 proteins have been previously identified as negative post-transcriptional regulators^28,30,35,54,55^, we found that MEX3A could also act as a positive regulator of the translation of some target RNAs, e.g. *Sdc4* (Fig. 3i).

**Fig. 4:**
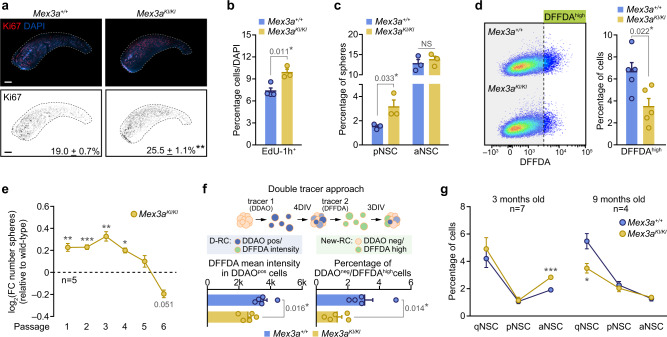
MEX3A maintains the balance between cell state transitions and stemness in NSC. **a** Immunohistochemistry for Ki67 (red) in *Mex3a*^*+/+*^ and *Mex3a*^*KI/KI*^ SEZ wholemount preparations (top panels). Binary images of Ki67 (black) for both genotypes and quantification of the percentage of Ki67^+^ cells in coronal sections of the SEZ (*n* = 4, *p*-value = 0.003) (bottom panels). **b** Quantification of the percentage of cells that incorporated EdU 1 h prior to sacrifice (EdU^+^-1h) in the SEZ of wild-type and MEX3A mutant mice. **c** Quantification of the percentage of neurospheres originated from isolated wild-type and MEX3A-deficient pNSC and aNSC (NS = 0.502). **d** Representative FACS plots of DFFDA staining in wild-type and KI homozygous cultures (left panel). Quantification of the percentage of DFFDA^high^ cells in *Mex3a*^*+/+*^ and *Mex3a*^*KI/KI*^ NSC cultures (right panel). **e** Self-renewal assay in wild-type and MEX3A-deficient NSC cultures. Data is represented as the log2 of the fold-change in the number of spheres relative to wild-type for each passage (dashed line) (*p*-value P1 = 0.003, P2 = 0.0003, P3 = 0.001, P4 = 0.011, P5 = 0.208, P6 = 0.051). **f** Schematic representation of the strategy using two sequential fluorescent tracers; D-RC: double retaining cell, New-RC: new retaining cell (top panel). Quantification of DFFDA intensity in DDAO^pos^ cells (left). Quantification of the percentage of newly generated semi-quiescent DDAO^neg^/DFFDA^high^ cells (right). **g** Quantification of the percentage of NSC populations by FACS in 3-months-old (p-value qNSC = 0.502, pNSC = 0.887, aNSC = 0.0001) and 9 months-old (*p*-value qNSC = 0.025, pNSC = 0.567, aNSC = 0.553) *Mex3a*^*+/+*^ and *Mex3a*^*KI/KI*^ mice. Graphs represent mean values and error bars show SEM. Exact *p*-values and the number of biologically independent samples (represented as dots) used are indicated in the graphs. ^*^p-value < 0.05, ^**^p-value < 0.01, ^***^p-value < 0.001 by unpaired two-tailed Student’s *t*-test. Source data are provided as a Source Data file. Scale bars: a, 500 μm.

Although *Mex3a*^*KI*/*KI*^ mice showed an evident reduction in body and brain size of around 20% accompanied by an expansion of the ventricular space (Supplementary Fig. 5d, e), we detected clear signs of hyperproliferation in the SEZ, with increased percentages of Ki67^+^ cells and of cells incorporating the thymidine analogue EdU administered 1 h before sacrifice (Fig. 4a, b). In order to directly ascertain the role of the MEX3A program at the transition to activation, we decided to check cycling probability among acutely isolated pNSCs, the stage in which *Mex3a* becomes first expressed. Clonal cultures of aNSCs and pNSCs^12^ showed that *Mex3a*^*KI*/*KI*^ shallow quiescent, but not fully activated NSCs exhibited a higher propensity to engage proliferation (Fig. 4c). In addition, *Mex3a*^*KI*/*KI*^ neurosphere cultures contained reduced numbers of DFFDA^high^ cells, also suggesting increased cell cycle entry (Fig. 4d). We have recently shown that slowly-dividing cells are largely responsible for the self-renewing properties of neurosphere cultures^12^. In accordance with this, MEX3A-deficient NSCs displayed an advantage during early passages in expanding cultures that was lost by passage 5 eventually leading to a reduced potential for neurosphere generation (Fig. 4e). Our data indicated that MEX3A could be acting as a molecular gatekeeper during NSC activation.

To explore the additional possibility that MEX3A could be playing a role in the return to quiescence, as a first approach we nucleofected wild-type neurosphere cells with plasmids containing *Mex3a-Gfp* or *Gfp* cDNA sequences and observed a great reduction in the incorporation of EdU in the MEX3A overexpressing cells (Supplementary Fig. 6a). To gain more insight, we used an in vitro assay of double fluorophore dilution, as we have described that slowly-dividing neurosphere cells can originate from fast-dividing ones through asymmetric division^12^. NSCs were first loaded with the far red-emitting tracer DDAO and, after 4 days, single cells dissociated from DDAO neurospheres were labeled with DFFDA and cultured for 3 more days (Fig. 4f). Doubly retaining cells (DDAO^pos^/DFFDA^high^) can be considered true semi-quiescent cells and the second tracer can be used to assess their proliferation as well as the generation of new slowly-dividing cells that originated from actively dividing cells, i.e. they diluted the first tracer (DDAO^neg^) but changed to a slower kinetics, hence retaining the second tracer (DDAO^neg^/DFFDA^high^)^12^. *Mex3a*^*KI*/*KI*^ primed-like DDAO^pos^ cells exhibited reduced DFFDA intensity (Fig. 4f), confirming an acceleration of the cell cycle kinetics in these semi-quiescent cells. In addition, MEX3A-deficient cultures had lower proportions of newly generated semi-quiescent DDAO^neg^/DFFDA^high^ cells suggesting that enhanced activation was coupled to a deficient return to slow cycling (Fig. 4f). In vivo, phenotypic analysis of our SEZ populations with cytometry in *Mex3a* wild-type and hypomorphic mice demonstrated that an increase in aNSCs at 3 months of age was followed by a loss of qNSCs at 9 months (Fig. 4g). The data together indicate that MEX3A binds to a set of quiescence-related mRNAs in activated NSCs that are needed for the eventual return to a more quiescent phase and hence very low levels of MEX3A ultimately leads to a reduction of the qNSCs pool in vivo.

### MEX3A is also required for the repression of a stemness-gliogenic signature at the onset of differentiation

**Fig. 5:**
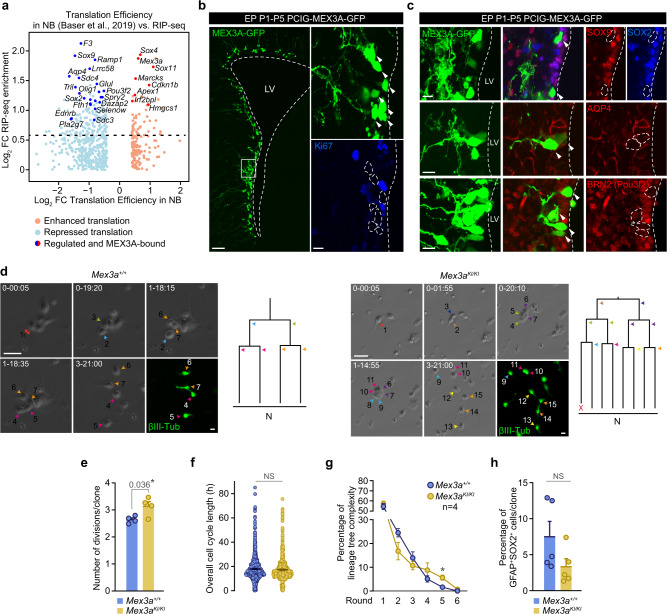
Dysregulation of the MEX3A targetome at the onset of differentiation leads to hyperproliferation. **a** Scatterplot comparing MEX3A RIP-seq enrichment data with translation efficiency data from SEZ and OB NBs^17^. Red dots represent genes with enhanced translation whereas blue dots represent genes with repressed translation. Dark red and blue dots label translationally regulated genes that are also bound by MEX3A. Four additional genes bound by MEX3A (*Apoe, Mmp14, Tpt1 and Zfp219*) showed uncoupled translation in NBs but differed between SEZ and OB, so they are not shown in the plot. **b** MEX3A-GFP signal (green) in the lateral ventricle of P5 mice 4 days after postnatal day 1 (P1) electroporation (EP) with PCIG-MEX3A-GFP (left). Ki67 (blue) expression in MEX3A-GFP^+^ electroporated cells (right). **c** Immunohistochemistry showing protein downregulation of MEX3A targets *Sox9* (red), *Sox2* (blue), *Aqp4* (red) and *Pou3f2* (BRN2, red) after overexpression of MEX3A-GFP. **d** Representative phase contrast images of *Mex3a*^*+/+*^ and *Mex3a*^*KI/KI*^ individual clones obtained by video time-lapse microscopy at different points (day-hour:minute) (left). Immunostaining for ßIII-tubulin (green) at the endpoint shows postmitotic cells with characteristic neuroblast morphology. Lineage trees derived from the clones shown in the pictures (right). Colored arrowheads indicate each division. X indicates cell death. **e** Quantification of the number of divisions per clone in *Mex3a*^*+/+*^ and *Mex3a*^*KI/KI*^ SEZ preparations (*n* = 4 mice). **f** Quantification of the overall cell cycle length (**h**) (NS = 0.184). **g** Percentage of clones that did undergo 1-to-6 rounds of divisions indicating lineage complexity of wildtype and MEX3A-deficient trees (^*^p-value = 0.032). **h** Percentage of GFAP^+^SOX2^+^ cells per clone in these trees (NS = 0.102). DAPI (blue) was used to counterstain nuclei. Arrowheads indicate positive cells. White boxes localize high-magnification inserts. Dashed lines mark the lateral ventricle (LV) and contour the nuclei of MEX3A-GFP^+^ cells. Graphs represent mean values and error bars show SEM. Exact *p*-values and the number of biologically independent samples (represented as dots) used are indicated in the graphs. ^*^p-value < 0.05, by unpaired two-tailed Student’s t-test. Source data are provided as a Source Data file. Scale bars: **b** 100 μm (insert, 15 μm); **b** and **c**, 15 μm; **d**, 30 μm.

*Mex3a* expression peaks at the NB stage and the in silico comparison of Baser et al. datasets with our RIP-seq data predicted that the set of MEX3A-bound transcripts belonging to the quiescent/gliogenic signature (e.g. *Olig1, Aqp4, Pou3f2, Ramp1, Sox9, Sox2, Sdc3, Sdc4*, or *Pla2g7*) would be translationally repressed in NBs (Fig. 5a). To evaluate the direct role of MEX3A in this repression we electroporated CAG-driven *Mex3a-Gfp* or *Gfp* cDNA-containing episomal plasmids into the LV of neonatal mice. We observed four days later that cells with exogenously increased expression of MEX3A had stopped cycling, as they were Ki67-negative (Fig. 5b), without affecting overall cell morphology and viability (Supplementary Fig. 6b–d). Cells overexpressing MEX3A exhibited remarkably reduced levels of glial NSC-associated SOX9, SOX2, AQP4, and *Pou3f2* (BRN2) levels (Fig. 5c). A member of the aquaporin family of membrane water channels, AQP4 is predominantly expressed in adult NSCs and astrocytes and its loss results in impaired subependymal NSC proliferation and self-renewal through unknown mechanisms^56,57^; SOX9 is a pro-gliogenic factor that sustains the generation and maintenance of subependymal NSCs^49^; transcription factor POU3F2 is required for hippocampal neurogenesis by an unknown mechanism^58^; SOX2 sustains the undifferentiated neural state and needs to be repressed at NB cell cycle exit^17,59–62^. Our results indicated that, as predicted, these NSC-associated transcripts can be translationally repressed by MEX3A.

The molecular data suggested that MEX3A repression of a set of gliogenic/stemness transcripts could be involved in NB specification and cell cycle exit. To directly test this possibility, we took advantage of a cell preparation model, in which cells isolated from the adult SEZ are kept in absence of growth factors, with the consequence that they maintain their intrinsic neurogenic nature allowing also for continuous live imaging by time-lapse video microscopy^63,64^. Employing this preparation, it is possible to track single NSCs and their progeny in real time and at a single cell level. Isolated SEZ neural progenitors were monitored for 5 days by time-lapse video-microscopy, acquiring brightfield pictures every 5 min. In addition, post-imaging immunocytochemistry revealed the nature of the generated progeny and software processing enabled the reconstruction of the lineage progression trees. The analysis of a total of 674 neurogenic lineage trees revealed a significant increase in the number of NB division rounds *per* clone in the cultures from *Mex3a* hypomorphs without changes in the duration of the cell cycle (Fig. 5d–f). Moreover, when lineage trees were analyzed independently, according to their rounds of division, a significant increase in the percentage of complex lineage trees (4-6 rounds of division) was observed in cultures derived from *Mex3a* hypomorphs (Fig. 5g), indicating increased neurogenic proliferation with a trending, but not significant effect on the founder population (Fig. 5h)^65^. The data indicated that reduced levels of MEX3A results in supernumerary rounds of divisions among NBs.

**Fig. 6:**
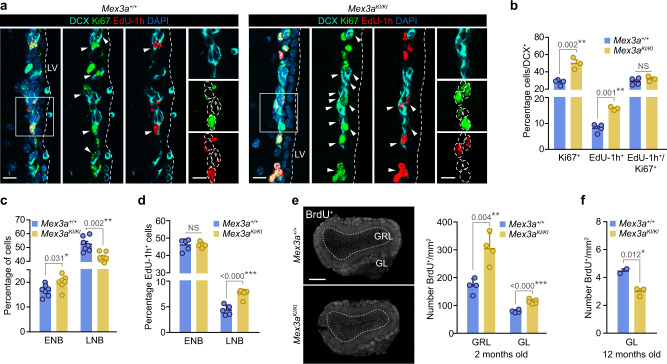
MEX3A is a gatekeeper for timely cell cycle exit of NB with an impact on adult neurogenesis. **a** Representative images of DCX (cyan) immunostaining in combination with Ki67 (green) and EdU-1h (red) in *Mex3a*^*+/+*^ and *Mex3a*^*KI/KI*^ adult SEZ sections. **b** Quantification of the percentage of Ki67^+^ cells, EdU-1h^+^ cells and the ratio EdU-1h^+^/Ki67^+^ in DCX^+^ NBs (NS = 0.311). **c** Quantification of the percentage of ENB and LNB populations by FACS. **d** Quantification of the percentage of EdU-labeled cells among NB populations after a one-hour pulse analyzed by FACS (NS = 0.895). **e** Immunohistochemistry for BrdU (white) in the OB of young adult mice (left panel). Quantification of the number of BrdU-28 days^+^ neurons reaching the granular (GRL) and glomerular (GL) layer of the OB in *Mex3a*^*+/+*^ and *Mex3a*^*KI/KI*^ mice injected at 2 months of age. **f** Quantification of the number of BrdU-28 days^+^ neurons reaching the GL of the OB in mice injected at 12 months of age. DAPI (blue) was used to counterstain nuclei. Arrowheads indicate positive cells. White boxes localize high-magnification inserts. Dashed lines mark the lateral ventricle (LV) and contour the nuclei of DCX^+^ cells. Graphs represent mean values and error bars show SEM. Exact *p*-values and the number of biologically independent samples (represented as dots) used are indicated in the graphs. ^*^p-value < 0.05, ^**^p-value < 0.01, ^***^p-value < 0.001 by unpaired two-tailed Student’s *t*-test. Source data are provided as a Source Data file. Scale bars: **a** 15 μm; **e** 500 μm; inserts, 10 μm.

Analysis of NBs in vivo revealed that the proportions of DCX^+^ cells that were also Ki67^+^ or had incorporated EdU after a 1 h-pulse were significantly increased in *Mex3a* hypomorphs. However, the ratio EdU/Ki67, which gives an idea of the relationship between the S phase relative to the total length of the cell cycle^66^, was preserved (Fig. 6a, b). Since a very large fraction of NBs exit the cell cycle after one or two rounds of cell division, the results indicated that MEX3A regulates cell cycle exit of NBs also in vivo. Proliferative early NBs and the far more numerous non-proliferative LNBs can be quantitated separately by flow cytometry after labeling with fluorescent EGF^12,41^. The analysis revealed that while EGFR^+^ NBs were overrepresented in the SEZ of young *Mex3a*^*KI*/*KI*^ mice, late EGFR^low/–^ NBs were significantly reduced (Fig. 6c). Strikingly, in *Mex3a* hypomorphs we found a higher labeling index in LNBs after a one-hour EdU pulse (Fig. 6d). Since the histological analysis indicated that NBs with reduced levels of MEX3A do not progress faster through the cell cycle, our data indicated an uncoupling between cell cycle exit and differentiation that would lead to additional cell divisions of late NBs while they downregulate EGFR expression. Our data indicated that MEX3A regulates molecular programs that are required to preserve an NSC-identity program that needs to be terminated when descendant progenitors commit to neuronal differentiation.

In order to analyze the combined effect of the dual MEX3A action on NSC long-term maintenance and neurogenesis, we set out to study the final neurogenic output to the OB with a classical birth-dating experiment. Two-month-old *Mex3a*^*+*/*+*^ and *Mex3a*^*KI*/*KI*^ mice were injected with BrdU every 2 h for 12 h and euthanized after 28 days^67^. MEX3A-deficient mice showed greater numbers of BrdU^+^ newly-generated neurons in the OB (Fig. 6e), a result that was in line with NBs undergoing more rounds of division. However, the same experiment in 12 months old mice revealed reduced neurogenesis in line with the progressive loss of quiescent NSCs (Fig. 6f and Fig. 4g). MEX3A is, therefore, a critical regulator of adult neurogenesis over the lifetime by its dose-dependent actions on translational adaptation at critical cell transitions.

## Discussion

Post-transcriptional control of existing coding transcripts allows for fast adaptation to evolving changes in a cell, like those associated to the onset of differentiation after cell cycle exit, which often require resetting the cell responsiveness machinery, repressing stemness programs, and/or increasing fate-specifying proteins to implement the right phenotype. However, post-transcriptional regulation is difficult to evaluate at the functional level as it frequently involves a large set of mRNAs whose compound effects can be very complex. In the present work we have identified the involvement of the RBP MEX3A in the regulation of adult neurogenesis and its role at specific cell transitions by proposing specific premises that were tested by in silico analysis of a RIP-seq experiment in comparison with published data sets of RNA transcription and translation in the subependymal lineage. These premises led to the identification of MEX3A-regulons that play specific roles at relevant cell transitions as revealed by the phenotype of mice with -drastically reduced levels of MEX3A. We show that MEX3A binds a significant number of transcripts of a stemness/quiescent-glial signature along the SEZ lineage. Our functional data indicate that MEX3A is required to preserve the capacity of aNSCs to return to a shallow quiescent state and that this capacity is important to avoid premature exhaustion of the deeply quiescent NSC pool. Later on, translational repression of this signature is required at the onset of neuronal differentiation, when MEX3A levels peak. The results also provide further insights into the molecular signatures associated to NSC quiescence and priming as well as to cell cycle exit at the onset of differentiation. Our data support a model in which the maintenance of NSC quiescence depends on a tight control over transcriptional programs at the mRNA level. MEX3A poses as an important regulator to coordinate the translation of a set of mRNAs in a timely and even dose-specific manner, thus providing a fine-tuned control over the lineage proteome to ensure accurate cell transitions.

Regulation of translation by RBPs in the brain has been studied during fetal development^21,22,68–74^, but the role of RBPs in adult NSCs and their cell derivatives has remained largely unexplored. Targets for MEX3 RBPs have been identified based on the presence of the *C. elegans* MRE consensus sequence and assayed individually^37,45,54^. Here we have used RIP-Seq analysis in a specifically developed mouse model to identify more than 200 MEX3A target transcripts, a potential MEX3A regulon in murine neurogenesis. From the proposed *C. elegans* MRE-based candidate mRNAs we could find at least 3 orthologs in our RIP-enriched list (*Ccng1*, *Sox2* and *Mex3a-d*)^45^, which might be indicative of some evolutionary conservation of the MEX3A’s function. However, the molecular mechanisms underlying target recognition and regulation by mammalian MEX3 proteins are yet to be elucidated. We have demonstrated that MEX3A acts as a post-transcriptional repressor like the nematode ancestral RBP, but it also shows positive effects on the translation of some targets. Compound dual effects on different targets of the regulon thus complicate a functional analysis of its role in cellular processes through the analysis of specific targets. Mammalian MEX3 proteins have acquired a C-terminal (RING) finger module with E3 ubiquitin ligase activity that is lacking in the nematode MEX-3^27,54^. The fact that different members of the MEX3 family display certain homologous relationships, their similar pattern of expression, and the dual regulatory role described for some of them^54,75–77^ suggest that vertebrate MEX3 proteins may share mechanisms of action and some functional redundancy. This is also supported by the binding of MEX3A binds to all *Mex3* mRNAs that we observed in NSCs which probably implies a tight feed-back regulation. In fact, MEX3B has also been shown to negatively regulate its own RNA to fine-tune its expression^77^. To date, MEX3 proteins have been reported to act through at least three different post-transcriptional regulatory mechanisms. MEX3D destabilizes human pro-apoptotic *BCL-2* mRNA by interacting with AU-rich elements (AREs) in its 3’UTR^78^. MEX3C promotes the deadenylation and degradation of the *MHC-I* mRNA^54,55^ through its association with the cytoplasmic deadenylation complexes and the ubiquitination of CNOT7, the main catalytic subunit of the CCR4-NOT deadenylation machinery. Finally, MEX3B up-regulates the expression of the pro-apoptotic protein BIM by interfering with the binding of a destabilizing Argonaute (Ago)-miR-92 complex to its target site in the 3’UTR^76^. All in all, MEX3 proteins stand out as highly versatile post-transcriptional regulators capable of acting through diverse molecular mechanisms.

## Methods

All experiments involving mice were performed at the animal housing facility (University of Valencia, Servei Central de Suport a la Investigació Experimental – SCSIE, Burjassot) in compliance with European Union 2010/63/UE and Spanish RD-53/2013 guidelines and under official veterinary supervision. All experimental procedures were approved by the Ethics Committee of University of Valencia (CEEA: 2015/VSC/PEA/00132 and 00133).

### Mouse strains and handling, genotyping and treatments

*Mex3a*^*tm1*(tdTomato-T2A-CreERT2)EBa^ reporter mice were generated by insertion of the tdTomato/T2A/Cre-ERT2/bGH-polyA construct in frame with the *Mex3a* start codon in exon 1, thus reducing expression of the *Mex3a* allele (hypomorphic allele)^40^. Heterozygous knock-in (KI) mice were used as reporter mice for *Mex3a* expression studies and henceforth named *Mex3a*^*+/KII*^. KI homozygous mice (*Mex3a*^*KI/KI*^) were used to analyze *Mex3a* deficiency while littermates not carrying the KI cassette (*Mex3a*^*+/+*^) were used as wild-type controls. *Mex3a*^*tm2*(3X-flag)EBa^ tagged MEX3A mice (*Mex3a*^*FLAG/FLAG*^) were generated by insertion of a 3X-FLAG sequence (aminoacidic sequence: DYKDHD-G-DYKDHD-I-DYKDDDDK) at the C-terminus of the *Mex3A* gene immediately 5’ of the stop codon using crispr/cas9 technology. Briefly, a 2X ribo-nuclear protein (RNP) complex consisting of tracrRNA, guide RNA and Cas9 protein was created as follows. tracrRNA and guide RNA (2.5 µl of each at 5 ng/µl) were mixed, briefly heated to 90 °C and allowed to cool to room temperature (RT). The guide/tracr mix was then added to 1.7 µl of purified Cas9 protein (60 ng/µl) and incubated for 10 min at RT to form the RNP. This RNP mix was stored at −80 °C until required. Immediately before electroporation, an equal volume of ssDNA repair template (20 ng/µl) was added to the RNP. Guide cRNA 19 was used in conjunction with ssDNA 19t, and guide cRNA 26 with ssDNA 26t (Supplementary Table 1). C57B6/j zygotes were combined with the RNP/ssDNA mix and electroporated using a NEPA 21 electroporator (Nepa Gene) with a 1.5 mm chamber, with the following conditions for: poring pulse (60 V, duration 1.5 ms, interval: 50 ms, number of pulses: 4, decay rate; 10%, positive polarity) and transfer pulse (5 V, length 50 ms, interval: 50 ms, number of pulses: 5, decay rate; 40%, positive/negative polarity). Electroporated zygotes were transferred into the oviduct of pseudopregnant recipient mice and allowed to develop to term. Offspring were analyzed by PCR to detect the presence of the 3X FLAG, followed by Sanger sequencing to confirm the presence of the correct sequence. All mice were bred and housed, under 12 h periods of light/darkness, room temperature of 20‒22 °C, 40–70% humidity and food and water provided *ad libitum*. Strains were maintained on a C57BL/6 J background and litters were weaned 21 days after birth. When using highly invasive techniques was inevitable, mice were first deeply anesthetized by intraperitoneal (i.p.) injection of a mixture of medetomidine (0.5–1 mg per kilogram of body weight) and ketamine (50–75 mg/kg) diluted in saline solution (0.09% NaCl). Genetically modified mice were genotyped by end-point Polymerase Chain Reaction (PCR) of genomic DNA (gDNA) extracted from an ear punch using the Phire Animal Tissue Direct PCR kit (Thermo Fisher, cat. no. F140WH). The specific primers for each PCR are also detailed in Supplementary Table 1. Both male and female mice (2–4 or 9–12 months old as indicated in each case) were included in all experiments. Experimental groups were designed by distributing littermates as evenly as possible among the groups. Administration of either 5-bromo-2´-deoxyuridine (BrdU) or 5-ethynyl-2´-deoxyuridine (EdU) in combination with different chase time points was used to evaluate proliferation of the SEZ populations and neurogenesis to the OB. For cell proliferation analysis, 2–4 months old mice received one intraperitoneal (i.p.) injection of EdU (50 mg/kg of body weight) (Life Technologies, cat. no. E10187) 1 h prior to sacrifice (EdU-1h). For neurogenesis to the OB, mice at 2 or 12 months of age were i.p. injected with 50 mg/kg of BrdU (Sigma, cat. no. B5002) every 2 h for 12 h (7 shots) and sacrificed 28 days after the last injection.

### Immunohistological analyses

Mice were deeply anaesthetized and transcardially perfused with 28 ml of saline buffer (0.9% NaCl) and 83 ml of 4% paraformaldehyde (PFA) in 0.1 M phosphate buffer pH 7.4 (PB) at a flow rate of 5.5 ml/min. Fixed brains were then post-fixed for 1 h by immersion in 4% PFA and vibratome-sectioned at 30–40 μm (adult) and 80 μm (postnatal) (Leica VT1000). For immunostaining, serially collected tissue slices were incubated in blocking buffer (10% Horse Serum (HS) and 0.1‒0.2% Triton^TM^ X-100 in 0.1 M PBS) for 1 h at RT and then with primary antibodies (rabbit anti-AQP4 (Sigma, cat no. HPA014784, 1:200); rabbit anti-DsRed (Takara, cat. no. 632496, 1:400); chicken anti-GFAP (Millipore, cat. no. AB5541, 1:800); mouse anti-ASCL1 (BD, cat. no. 556604, 1:100); chicken anti-DCX (Abcam, cat. no. ab153668, 1:500); rabbit anti-DCX (Abcam, cat. no. ab18723, 1:1000); goat anti-DCX (Santa Cruz, cat. no. sc-8066, 1:300); mouse anti-NeuN (Millipore, cat. no. MAB377, 1:200); goat anti-POU3F2/BRN2 (Santa Cruz Biotechnology; cat no. sc-6029, 1:50); mouse anti-S100β (Sigma, cat. no. S2532, 1:500); rabbit anti-Ki67 (Abcam, cat. no. ab15580, 1:300); goat anti-SOX2 (R&D, cat. no. AF2019, 1:600); rabbit anti-SOX9 (Abcam, cat. no. AB184547, 1:1000); rabbit anti-bIII-TUBULIN (Sigma, cat. no. T2200, 1:300); rat anti-BrdU (Abcam, cat. no. ab6326, 1:800)) diluted in blocking solution overnight at 4 °C. After washing with 0.1 M PBS, slices were incubated with fluorescent-labeled secondary antibodies (Alexa Fluor® 488 Donkey Anti-chicken (Jackson ImmunoResearch, 703-545-155, 1:800); Alexa Fluor® 488 Donkey Anti-mouse (Molecular Probes, cat. no. A21202, 1:800); Alexa Fluor® 488 Donkey Anti-rabbit (Jackson ImmunoResearch, cat. no. 711-547-003, 1:800); Alexa Fluor® 647 Donkey Anti-chicken (Jackson ImmunoResearch, cat. no. 703-606-155, 1:800); Alexa Fluor® 647 Donkey Anti-goat (Molecular Probes, cat. no. A21447, 1:800); Cy3 Donkey anti-chicken (Jackson ImmunoResearch, cat. no. 703-165-155, 1:800); Cy3 Donkey anti-mouse (Jackson ImmunoResearch, cat. no. 715-165-151, 1:800); Cy3 Donkey anti-rabbit (Jackson ImmunoResearch, cat. no. 711-165-152, 1:800); Cy3 Donkey anti-rat (Jackson ImmunoResearch, cat. no. 712-165-153, 1:800)) for 1 h at RT. Finally, nuclei were counterstained with 4′,6-diamidino-2-phenylindole (DAPI, 1 µg/ml) for 5 min and sections were mounted with FlourSave^TM^ Reagent (Calbiochem, cat. no. 345789). For BrdU detection, sections were pre-incubated in 2 N HCl for 20 min at 37 °C and neutralized in 0.1 M sodium borate (pH 8.5) prior to incubation with blocking solution. EdU detection was carried out using the Click-iT^TM^ EdU Alexa Fluor^TM^ 555 Imaging Kit (ThermoFisher, cat. no. C10338). For staining of SEZ whole-mount preparations, samples were incubated with primary antibodies for 48 h and 0.1% Triton^TM^ X-100 was used during washing steps. Images were acquired using an Olympus FV10i confocal microscope (405, 458, 488 and 633 nm lasers; Olympus) with Olympus FV10i-SW (v2.1, Olympus) and with a Zyla 4.2 sCMOS camera (Andor) in a Nikon ECLIPSE Ni-U microscope (Nikon) coupled to a LED pE-300-W unit (CoolLED) with NIS-Elements BR (v5.11, Nikon). The area of brain sections from 2–4 months old mice was quantified using ImageJ software. For brain and lateral ventricle area estimation, DAPI stained series containing 4 coronal sections (1 hemisphere) of the anterior horn of the lateral ventricles (Bregma antero-posterior coordinates +1.10, +0.74, +0.38 and +0.02 mm) were panoramically imaged. The whole hemisphere and the lateral ventricle were outlined and measured for each stereotaxic coordinate and the mean area was represented. Cell populations were manually counted using the ImageJ/Fiji software (v1.57 f) and data was obtained as a percentage of positive cells relative to a subpopulation or to total DAPI labeled cells in the lateral ventricle wall. Neurogenesis to the OB was evaluated as the number of BrdU^+^ cells that reached the granular layer (GRL) and the glomerular layer (GL) of the main olfactory bulb after 28 days. Between 7 and 10 coronal sections in the medial OB (Bregma AP coordinates +5 to +4 mm) were analyzed. The total number of BrdU^+^ cells in the GL were manually counted under a Nikon ECLIPSE Ni-U fluorescence microscope (Nikon) and normalized to the GL area previously calculated in a BX61 motorized microscope (Olympus). For the GRL analysis, sections were photographed in an Olympus FV10i confocal microscope and data was obtained as the number of BrdU^+^ cells *per* total imaged area.

### In vivo postnatal electroporation

PCIG-GFP (plasmid #11159, Addgene) deposited by Connie Cepko, Harvard Medical School^79^, was used as a control empty vector and as a backbone to generate PCIG-Mex3a-GFP. PCIG-GFP vector contains the chicken 5-actin promoter, an internal ribosome entry site (IRES), a multicloning site (MCS) and the coding sequence of GFP. The *Mex3a* coding sequence (NM_001029890.3) was subcloned into PCIG-GFP by PCR introducing XbaI and EcoRI restriction sites for its introduction in the MCS. In vivo postnatal electroporation was performed as described previously^80^. Briefly postnatal day 1.5 (P1.5) wild-type mouse pups were deeply anesthetized by isoflurane inhalation. Once the pedal reflex was lost, ≈1 µl of a 2 µg/µl plasmid solution was injected into one of the lateral ventricles of the pup. 5 pulses of 95 V were delivered, spaced by 950 ms, using forcep electrodes (Platinum Tweezertrode 5 mm Diameter, Btx) with a 45° degree to target the subpalial SEZ and a square wave electroporator (ECM 830 Square Wave Electroporation System, Btx). Pups were reanimated in a heating pad for a few minutes and returned to the mother’s cage until perfusion at P5.5.

### SEZ neurosphere cultures

Adult subependymal NSC cultures were obtained from 2–4 months old mice as previously described^81^. Briefly, both SEZs from each brain were dissected, minced and enzymatically digested in 1 ml of 12 units/ml papain (Worthington Biochemical Corporation, cat. no. LS003120) in enzymatic solution (0.2 mg/ml EDTA (Sigma, cat. no. E6511) and 0.2 mg/ml L-cysteine hydrochloride (Sigma, cat. no. C8277) in Earle’s Balanced Salt Solution (EBSS) (Gibco, cat. no. 24010-043)) for 30 min at 37 °C, followed by gentle mechanical dissociation. Primary cells were grown in NSC medium supplemented with 20 ng/ml EGF (Gibco, cat. no. 53003-018), 10 ng/ml bFGF (Sigma, cat. no. F0291) and 1X B27 (Thermo Fisher, cat. no. 12587010) for 7–10 days at 37 °C in a 5% CO_2_ humidified incubator. For culture passage and bulk expansion, 4–6 days secondary neurospheres were dissociated with Accutase® solution (Sigma, cat. no. A6964) and subcultured at 10,000 viable cells/cm^2^. The capacity of individual cells to generate new neurospheres in sequential Neurosphere Formation Assays (NFA) with the same cultures at different passages (passages 1–6) was used to assess alterations of NSC self-renewal. In order to do so, individual cells were seeded at pseudo-clonal density (5 cells/μl) and 5–6 days neurospheres were manually counted on a Nikon ECLIPSE TE2000-S inverted microscope with phase contrast optics (Nikon). Data was represented as log2 of the fold-change in the number of spheres. To assess protein levels of MEX3A potential targets by immunodetection of surface antigens by flow cytometry, 3‒4 DIV neurospheres were manually dissociated by carefully pipetting several times with a p200 tip. Individual cells were incubated with 100 μl of the specific fluorescent-labeled primary antibodies (human anti-SDC4-APC (Miltenyi, cat. no. 130-109-831, 1:200); rabbit anti-AQP4 (Sigma, cat. no. HPA014784, 1: 150)) in flow cytometry blocking buffer at 4 °C for 30 min. Immunostained samples were analyzed in an LSR-Fortessa cytometer (350, 405, 488, 561 and 640 nm lasers, Becton Dickinson) with FACSDiva (v8.0.2, BD). DAPI (0.1 µg/ml) was added to exclude dead cells from the analysis. An additional fixation step with 100 µl of Cytofix/Cytoperm^TM^ solution (BD Bioscience, cat. no. 554722) (20 min at 4 °C) and incubation with an A647-labeled secondary antibody was used (Alexa Fluor® 647 Donkey Anti-rabbit (Molecular Probes, cat. no. A31573, 1:300)) (30 min at 4 °C) to detect AQP4. FACS analyses were performed in FlowJo (v11, BD).

### SEZ culture preparation and time-lapse microscopy

Adult SEZ-derived NSCs were isolated from 2-3 months-old *Mex3a*^*+/+*^ or *Mex3a*^*KI/KI*^ mice and cultured following the method described in^62^. Briefly, the tissue was enzymatically dissociated in 0.7 mg/ml hyaluronidase (Sigma-Aldrich, cat. no. H3884) and 1.33 mg/ml trypsin (Sigma-Aldrich, cat. no. T9201) in Hanks’ Balanced Salt Solution (HBSS; Invitrogen, cat. no. 14065-049) with 2 mM glucose at 37 °C for 30 min. After several washes to remove cell debris, the cell pellet was resuspended in culture medium containing DMEM/F12 Glutamax (Invitrogen, cat. no. 31966021) supplemented with B27 (Invitrogen, cat. no. 17504-044), 2 mM glutamine (Gibco, cat. no. 25030024), 100 units/ml penicillin-streptomycin (Invitrogen, cat. no. 15140148) and 8 mM HEPES, and cells were plated on poly-D-lysine (Sigma, cat. no. P0899) coated coverslips at a density of 200–300 cells/mm^2^. Time-lapse video microscopy and single cell tracking^62,82–84^ of adult SEZ cultures was performed with a Nikon TE-2000 microscope at a constant temperature of 37 °C and 8% CO_2_. Phase contrast/Bright field images were acquired every 5 min for 5 days using a 20x phase contrast objective (Nikon), a ZYLA from ANDOR camera and a NIS-element from NIKON software. For subsequent single cell tracking a self-written computer program (TTT) was employed^85^. Movies were assembled using Image J 1.42q (National Institute of Health, USA) software and are played at speed of 1-3 frames per second.

### SEZ dissociation, flow cytometry analysis and sorting of in vivo populations

SEZ populations were analyzed as previously described^12,41^. Briefly, SEZs were minced and enzymatically digested using the Neural tissue dissociation kit (T) (Miltenyi, cat no. 130-093-231) following the instructions of the manufacturer in a gentleMACS Octo Dissociator with heaters (Miltenyi). Digestion was quenched with 3 ml of 100 μg/ml trypsin inhibitor (Sigma, cat no. T6522) and the digested pieces were gently mechanically dissociated and filtered through a 40 μm nylon filter. The eluted fraction was resuspended in 100 μl of flow cytometry blocking buffer (0.1% Glucose, 10 mM HEPES, 2 mM EDTA and 0.5% BSA in HBSS) with the specific primary antibodies and reagents (anti-CD45-BV421 (BD Bioscience, cat. no. 563890, 1:200); anti-TER119-BV421 (BD Bioscience, cat. no. 563998, 1:200); anti-CD31-BV421 (BD Bioscience, cat. no. 563356, 1:100); anti-O4-biotin (Miltenyi, cat. no. 130-095-895, 1:30); BV421 streptavidin (BD Bioscience, cat. no. 563259, 1:300); anti-CD24-PerCP-Cy5.5 (BD Bioscience, cat. no. 562360, 1:300); anti-CD24-BB700 (BD Bioscience, cat. no. 746122, 1:300); anti-CD9-APC-VIO770 (Miltenyi, cat. no. 130-102-384, 1:20); anti-GLAST-APC (Miltenyi, cat. no. 130-095-814, 1:20), EGF-488 (Molecular Probes, cat. no. E13345, 1:300)) at 4 °C for 30 min. After washing, labeled samples were resuspended in 0.5 ml of blocking buffer for analysis in an LSR-Fortessa cytometer (350, 405, 488, 561 and 640 nm lasers, Becton Dickinson) with FACSDiva (v8.0.2, BD). DAPI (0.1 µg/ml) was added to exclude dead cells from the analysis. To assess proliferation in EdU-injected mice, samples were fixed with 100 µl of Cytofix/Cytoperm^TM^ solution (BD Bioscience, cat. no. 554722) for 20 min at 4 °C and developed using the Click-iT^TM^ Plus EdU Alexa Fluor^TM^ 555 Flow Cytometry Assay Kit (ThermoFisher, cat. no. C10638) following manufacturer’s instructions. FACS analyses were performed in FlowJo (v11, BD). See Supplementary Fig. 7 for gating strategy. For pNSC and aNSC sorting, the SEZ of 2 mice were processed together as described above. After enzymatic dissociation and filtering, cells were incubated with Myelin Removal beads (Miltenyi, cat. no. 130-096-731) and passed through a previously equilibrated MS column (Miltenyi, cat. no. 130-042-401) on a MidiMACS^TM^ magnetic separator (Miltenyi) following manufacturer’s guidelines. Then, eluted fractions were collected, pelleted (300xg, 10 min) and processed for fluorescent labeling as described above. pNSC and aNSC cell fractions were isolated in a BD FACSAria III (350, 405, 488, 561 and 640 nm lasers) using a 100 μm nozzle at 20 psi. DAPI (0.1 µg/ml) was added to exclude dead cells from the sorting. 350 cells were sorted into 200 μl of NSC medium supplemented with 20 ng/ml EGF (Gibco, cat. no. 53003-018), 10 ng/ml bFGF (Sigma, cat. no. F0291) and 1X B27 (Thermo Fisher, cat. no. 12587010) for neurosphere formation. The number of pNSC and aNSC-derived neurospheres was assessed after 7 days.

### Evaluation of cell proliferation dynamics in vitro

For proliferation assessment after MEXA overexpression, 4-5 days wild-type secondary neurospheres were disaggregated with Accutase® solution (Sigma, cat. no. A6964), mixed with either PCIG-GFP or PCIG-Mex3a-GFP (1 μg per million of single cells) plus 100 μl of supplemented nucleofection solution from the Mouse Neural Stem Cell Nucleofector^TM^ Kit (Lonza, cat. no. VPG-1004) and immediately nucleofected with a Nucleofector 2b device (Amaxa) with G-013 preset program. Cells were recovered overnight in NSC medium supplemented with 20 ng/ml EGF (Gibco, cat. no. 53003-018), 10 ng/ml bFGF (Sigma, cat. no. F0291) and 1X B27 (Thermo Fisher, cat. no. 12587010). The next day, cells were collected, washed and seeded on Matrigel^TM^ (Corning®, cat. no. 354230) coated 96-well plates at 5000 cells per well in supplemented NSC medium. After 18–20 h, cells received a pulse of 10 μM EdU (Click-iT^TM^ EdU Alexa FluorTM 555 Imaging Kit; ThermoFisher, cat. no. C10338) for 1 h at 37 °C and then were fixed with 2% PFA for 20 min at RT. Next, cells were immunostained with chicken anti-GFP antibody (Aveslabs, cat. no. GFP-1020, 1:500) overnight at 4 °C and then with Alexa Fluor® 488 Donkey Anti-chicken secondary antibody (Jackson ImmunoResearch, 703-545-155, 1:800) for 30 min at RT. After washing the secondary antibody, the presence of EdU was developed using the Click-iT^TM^ Plus EdU Alexa FluorTM 555 Flow Cytometry Assay Kit (ThermoFisher, cat. no. C10638) following manufacturer’s instructions. Finally, nuclei were counterstained with 1 µg/ml DAPI for 2 min at RT. Fifty 12-bit images per well were captured for each channel with a x20 objective in an INCell Analyzer 2000 automated microscope (GE Healthcare). Unbiased analysis was performed with ImageJ-FIJI (version 1.53 f, NIH) as follows: nuclei were segmented with the StarDist algorithm^86^, the resulting regions-of-interest were filtered to remove particles smaller than 50 square pixels and finally redirected to the EdU and GFP channels to measure their respective mean gray value. A threshold level of 150 was established for both channels to score cells as positive. For cell proliferation evaluation by dilution of fluorescent cell tracers, 1 million single cells were washed with DPBS (200xg, 10 min) and incubated with 2 μg/ml of CellTrace^TM^ Oregon Green^TM^ 488 Carboxy-DFFDA-SE (Thermo Fisher, cat. no. C34555) in DPBS for 7 min at 37 °C in a thermostatic bath in the dark. Probe excess was removed with washing medium (200xg, 10 min) and loaded cells were seeded in complete medium for 4 days at 37 °C in a 5% CO_2_ humidified incubator. Heterogeneous dilution of the tracer within the culture defined label retaining cells (DFFDA^high^) and highly proliferative cells that were analyzed in a LSR-Fortessa cytometer (Becton Dickinson) with 350, 405, 488, 561 and 640 nm lasers. Note that to compare DFFDA populations between genotypes we established an intensity threshold that was applied for all experiments. When two tracers were used to evaluate return to quiescence^41^, 2 μg/ml of CellTrace^TM^ Far Red DDAO-SE (Thermo Fisher, cat. no. C34553) was loaded first and cells were cultured for 4 days. Then, these spheres were split, reloaded with 2 μg/ml of CellTrace^TM^ Oregon Green^TM^ 488 Carboxy-DFFDA-SE and plated in complete medium for 3 more days. Finally, secondary neurospheres were dissociated and the fluorescence intensity of each cell tracer was measured in a LSR-Fortessa flow cytometer and analyzed in FlowJo (v11, BD).

### RNA extraction and RT-qPCR expression analysis

RNA from NSC cultures was extracted and DNase-treated with the Maxwell® 16 LEV simplyRNA Tissue Kit (Promega, cat. no. AS1280) following the manufacturer’s guidelines. Then, it was quantified using the Qubit^TM^ RNA HS Assay Kit (Thermo Fisher, cat. no. 32852) in a Qubit^TM^ Fluorometer (Thermo Fisher) and 0.5‒1 µg was reverse transcribed using the PrimeScript RT reagent kit (Takara, cat. no. RR037A) in the presence of both 50 pmol random hexamers and 25 pmol oligo-dT primer (final volume of 20 µl). Gene expression was analyzed by reverse transcription quantitative PCR (RT-qPCR) in a Step One Plus detection system (Applied Biosystems) using 5‒15 ng of cDNA and 20 pmol of specific SYBR® Green primers (Supplementary Table 1; SYBR® Premix Ex Taq^TM^ Kit (Takara, cat. no. RR420) in a touch-down (TD) PCR with annealing temperature 65TD60 (descending −0.5 °C every cycle) and with an additional extension step at 72 °C. Primers to amplify each one of the *Mex3* family members (*Mex3a*, *Mex3b*, *Mex3c* and *Mex3d*) were designed after Clustal Omega alignment (EMBL-EBI) of their reference sequences, by including a paralog specific nucleotide in the 3’-end of each primer. Specificity of the obtained amplicons was verified by Sanger sequencing. Expression levels were assessed by relative quantification using constitutive expression of *Gapdh* (Mm99999915_g1), *18* *S* (Hs99999901_s1) and *Rpl32* (Mm02528467_g1) as housekeeping endogenous controls and normalized to a reference sample.

### Protein extraction and immunodetection by Western Blot

3 DIV NSC cultures were harvested and lysed with cold RIPA buffer (50 mM Tris-HCl pH 8, 150 mM NaCl, 1 mM MgCl2, 1% NP-40, 0.5% SDS, 0.1% sodium deoxycolate, 1 mM EDTA) supplemented with fresh cOmplete^TM^ protease inhibitor cocktail (Roche, cat. no. 11836153001), 1 mM sodium orthovanadate and 1 mM sodium fluoride (NaF), thoroughly vortexed and placed on ice for 30 min – 1 h. Samples were sonicated with a UP100H ultrasonic processor (Hielscher, Ultrasound Technology) and protein concentration of the lysates was quantified using the Pierce^TM^ BCA Protein Assay Kit (Thermo Scientific, cat. no. 23225) according to manufacturer’s guidelines. 10 µg of total protein in Laemmli buffer were loaded into a 10‒12% polyacrylamide electrophoresis gel (SDS-PAGE) and transferred onto a PVDF membrane (Trans-Blot® Turbo^TM^ Mini PVDF Transfer Packs) (Bio-Rad, cat. no. 1704156) using the Trans-Blot® Turbo^TM^ Transfer System (BiSDCo-Rad). For protein immunodetection, membranes were first blocked with 5% milk in TBS-T (0.1% Tween-20 in TBS recipe) for 1 h at RT and then incubated with specific primary antibodies (goat anti-FLAG (Abcam, cat. no. ab1257, 1:1000); rabbit anti-MEX3A (Abcam, cat. no. ab79046, 1:1000); mouse anti-alphaTUBULIN (Sigma, cat. no. T9026, 1:10000) in blocking buffer overnight at 4 °C. After washing thoroughly with TBS-T, HRP-conjugated antibodies (HRP Goat anti-mouse (Dako, cat. no. P0447, 1:5000); HRP Goat anti-rabbit (Santa Cruz, cat. no. sc-2004, 1:5000); HRP Rabbit anti-goat (Dako, cat. no. P0449, 1:5000) were incubated for 1 h at RT and developed with either Western Lightning Plus-ECL (PerkinElmer, cat. no. NEL103001EA) or SuperSignal^TM^ West Femto Maximum Sensitivity Substrate (Thermo Scientific, cat. no. 34095). Images were acquired in a Mini HD 9 chemiluminescence imaging system (Uvitec, Cambridge) and quantified with ImageJ software. Protein levels were normalized to loading controls and represented as fold change relative to wild-type. Samples from MEX3A immunoprecipitation were directly denaturalized in Laemmli buffer, loaded into a 10% SDS-PAGE gel and transferred onto a PVDF membrane as described above.

### MEX3A Immunoprecipitation (IP)

In order to identify MEX3A target RNAs, endogenous MEX3A was immunoprecipitated in FLAG-tagged NSC cultures (*Mex3a*^*FLAG/FLAG*^). Wild-type cultures from the same background that did not express the knock-in FLAG sequence were used as controls for nonspecific binding. 2-3 biological replicates for both the FLAG-tagged and the wild-type cultures were used, and the IP was performed both with crosslinked and non-crosslinked samples. That is because native (without crosslinking) conditions seemed to immunoprecipitate MEX3A better (see Supplementary Fig. 3a), but crosslinking may maintain more interactions. Thus, in order to obtain stronger results both IPs were carried out in parallel, and all IPs were considered as independent experiments for RNA analysis. 3 DIV neurospheres from passage 5‒6 *Mex3a*^*FLAG/FLAG*^ cultures (5 – 6 million cells) were collected and crosslinked with 0.5% formaldehyde (Thermo Fisher, cat. no. 28908) in 0.1 M DPBS for 5 min at RT with soft agitation. Crosslinking was stopped with 0.25 M glycine for 4 min and washed twice with ice-cold DPBS. Next, pellets were lysed on ice with cold RIPA buffer (50 mM Tris-HCl pH 8, 150 mM NaCl, 1 mM MgCl_2_, 1% NP-40, 0.5% SDS, 0.1% sodium deoxycholate, 1 mM EDTA) supplemented with protease inhibitors (cOmplete^TM^ protease inhibitor cocktail, 1 mM sodium orthovanadate and 1 mM sodium fluoride) and 200 U/ml RNase inhibitor RiboLock (Thermo Fisher, cat. no. EO0381) and sonicated in a UP100H ultrasonic processor (Hielscher, Ultrasound Technology) (30% amplitude with 0.5 s pulses for 5 min). Finally, samples were vortexed and centrifuged at full speed so that supernatants were used for immunoprecipitation (IP). For IP in native conditions (without crosslinking), neurospheres were directly lysed after washing and samples were processed following the same protocol as the crosslinked ones. Prior to the IP, 10 µg (1:5) of mouse anti-FLAG antibody (Sigma, cat. no. F1804) were incubated with 50 µl of magnetic Protein G Dynabeads (Invitrogen, cat. no. 10003D) for 2 h at RT on a wheel. After washing the excess antibody, antibody-conjugated beads were washed twice with 0.2 M triethanolamine pH 8.2 and crosslinked with fresh 20 mM dimethyl pimelimidate dihydrochloride (DMP) in triethanolamine for 30 min at RT on a wheel. Crosslinking was stopped with 0.05 M glycine for 15 min and beads were washed and transferred to RIPA buffer supplemented with protease and RNase inhibitors. Fresh lysates were precleared with 20 µl of Protein G Dynabeads for 30 min at 4 °C on a wheel. 5% of the precleared sample was stored as input and the remaining lysate was incubated overnight with the antibody-conjugated beads at 4 °C on a wheel. Immunoprecipitated RNPs were washed with RIPA buffer and processed for RNA or protein extraction while 5% of the unbound fraction was stored for western-blot analysis.

### Protein extraction for western-blot and LC-MS/MS protein identification of IP samples

After IP, beads were incubated with Laemmli buffer (62.5 mM Tris-HCl pH 6.8, 10% glycerol and 1% SDS) in agitation for 20 min at 65 °C to elute immunoprecipitated proteins. 50 mM DTT and bromophenol blue were added after elution and samples (including inputs and unbound samples) were boiled for 5 min at 98 °C. In order to specifically detect MEX3A by western-blot after the IP and not mouse immunoglobulins that could have potentially immunoprecipitated, a goat anti-FLAG antibody (Abcam, cat. no. ab1257; 1:1000) was used.

### RNA extraction for RIP-seq analysis of IP samples

After IP, dynabeads were incubated with 70 µg Proteinase K (Roche, cat. no. 03115879001) (prepared in 200 mM Tris-HCl pH 7.5, 100 mM NaCl, 10 mM EDTA, 1% SDS and 200 U/ml Ribolock) in agitation for 30 min at 42 °C and then for 30 min at 60 °C. Inputs and native IP samples were also treated with Proteinase K following the previous guidelines. Total RNA was extracted with RNeasy® Plus Micro kit (Qiagen, cat. no. 74034) following the manufacturer’s instructions. Note that DNA was removed by gDNA eliminator mini spin columns and the Appendix D modification was followed to ensure purification of total RNA including small RNAs (<200 bp). Finally, RNA concentration of the inputs, which was similar between samples and replicates (24.1 ± 5.0 ng/μl in WT vs 27.8 ± 5.1 ng/μl in FLAG), was verified in a 2100 Bioanalyzer (Agilent) using the total RNA 6000 Pico Kit (Agilent, cat. no. 5067-1513). This allowed us to use Mock IP as reference to account for nonspecific antibody binding to RNA fragments, since in the recent literature it has been shown that Mock IP performs equal, if not better, than Input to detect true IP signals^87^.

### RIP-seq library preparation, sequencing and quality control

8 µl of RNA from RIP (total of 10 samples, including both cross and not-crosslinked) were used to construct libraries for sequencing with the TruSeq® Stranded mRNA Library Prep Kit (Illumina, cat. no. 20020594), using the Dual index Nextera XT v2 Set A Adapters (Illumina, cat. no. FC-131-2001). Since RNA was already enriched due to IP, no purification and fragmentation steps were needed for library preparation, thus only random hexamers and not poly-T oligos were used during the process. Then, libraries were sequenced in a NextSeq 500 system (Ilumina) to an output of 566 (1x75nt) million reads. Total reads were analyzed with FastQC (v0.11.7) and visually inspected to make sure each sequenced sample met the adequate quality standards. Cutadapt (v1.16)^88^; and Trimmomatic (v0.36)^89^ were used to clip, filter and remove certain portions of reads or even entire reads. The Illuminaclip function was used to remove Illumina adapters. The SLIDINGWINDOW function was applied to filter out reads shorter than 20 bases and the MINLEN function was used to discard reads with an average quality of less than 28. A second round of inspection with FastQC was carried out after the filtering steps to ensure that the filters were applied properly. Filtered reads were mapped to the mouse genome with HISAT2 (v2.1.0)^89,90^, using the *Mus musculus* GRCm38 (mm10) genome as reference. Alignment files and associated coverage tracks were visually inspected with the genome browser IGV (http://software.broadinstitute.org/software/igv/). The similarity of biological replicates was checked with correlation analysis (see Supplementary Fig. 3b). For that, a matrix of read coverages along the entire genome was generated with all alignment files with the function multiBamSummary from the deepTools2 Python package (v3.1.1)^91^. This function takes the genome annotation and splits it into 10 kilobase bins in which the number of reads found in each alignment dataset is counted. Next, a Spearman correlation matrix heatmap plot was generated with the plotCorrelation function of the same package. Correlation values were overall high between samples, which pointed to the absence of major batch effects.

### Bioinformatic analysis of RIP-seq datasets

A matrix with raw read counts for each sample was generated with the featureCounts tool from the R package Rsubread (v1.6.2)^92^, using the mouse genome version M16 (Ensembl 91) annotation as a reference. Of those, 62% mapped to protein-coding genes, and 13% mapped to ribosomal RNA. In order to normalize the gene expression matrix so read counts could be directly comparable between samples, the DESeq2 (v1.16.1) package was used with the raw count expression matrix as input and with a 0.3 non-differential contig count quantile threshold. Once normalized, DESeq2 was used to perform a differential count analysis between RIP and wild-type control samples. The output lists were inspected and genes with more than 0.58 log2 FC (fold-change FC > 1.5 times) and a FDR value (False Discovery Rate) <0.05 were considered as positively enriched in the RIP compared to wild-type background. The lists of enriched genes and their respective p-values were used as inputs for the STRING (https://string-db.org/) analysis tool to generate networks and lists of Biological Process-related gene ontology (GO) terms. A threshold of FDR < 0.05 was selected to pick the significant terms. All heatmaps were generated using the ComplexHeatmap (v1.10.2)^93^ and ggplot2 (v3.0.0)^94^ packages in R. For expression heatmaps, the Spearman rank correlation distance was used as the base for the hierarchical clustering of genes. Normalized average density plots around genomic features were calculated with the R and Python software ngs.plot (v2.61)^95^ using indexed alignment files as inputs and the internal mm10 database annotation as reference. The statistical robustness parameter, which filters out 0.5% of genes with the most extreme (high and low) count values, was applied to all calculations. The 3’UTR region DNA sequences of 196 MEX3A-bound genes were extracted in fasta format and used as input for the FIMO tool from the MEME Suite (v4.11.4)^96^. A MEX3 consensus binding sequence motif (MRE, MEX3 recognition element) and its variants (DKAGN(0-8)THTA) (see Supplementary Fig. 3f)^45^, were used as the user-defined IUPAC string required by the FIMO tool as reference library. As a control group, a set of 200 genes with close-to-background levels of MEX3A RIP-seq enrichment were used for FIMO analysis using the same parameters, and the results were compared with MEX3A-bound genes using an unpaired t-test. The overlap between two lists of genes was represented as a Venn Diagram and analyzed at two levels: 1) the representation factor, and 2) the probability of finding the overlap. The representation factor is the number of overlapping genes divided by the expected number of overlapping genes drawn from two independent lists. A representation factor > 1 indicates more overlap than expected of two independent lists, a representation factor <1 indicates less overlap than expected, and a representation factor of 1 indicates that the two lists share the number of genes expected for independent lists. The probability is then calculated as the exact hypergeometric probability as C(D, x) * C(N-D, n-x) / C(N,n), being: x = # of genes in common, *n* = # of genes in list 1, D = # of genes in list 2, *N* = total genes, C(a,b) is the number of combinations of a things taken b at a time, the representation factor = x / expected # of genes, expected # of genes = (n * D)/N.

### Protein identification by LC-MS/MS

Proteins that co-immunoprecipitated with MEX3A were identified by liquid chromatography coupled to tandem mass spectrometry (LC‒MS/MS) in a DDA (data dependent acquisition). Protein samples from crosslinked IPs were digested with sequencing grade trypsin (Promega, cat. no. V5111)^97^, vacuum dried and resuspended in 2% acetonitrile (ACN), 0.1% trifluoroacetic acid (TFA). One third of the sample was loaded onto a Eksigent NanoLC trap column (ChromXP C18, 3 μm 120 Å, 350 μm x 0.5 mm) (SCIEX, cat. no. 5016752) and desalted with 0.1% TFA at 3 μl/min for 5 min. Then, peptides were loaded onto an analytical column (3 μm particles size C18-CL, 75 µm x 12 cm) (Nikkyo Technos Co®) equilibrated in 5% ACN, 0.1% formic acid (FA). Peptide elution was performed by applying a linear gradient of solvents A (0.1% FA in water) and B (0.1% FA in ACN) from 5% to 40% of solvent B in A at a constant flow rate of 300 nl/min over 90 min. Finally, peptides were analyzed in a nanoESI qQTOF mass spectrometer (5600 TripleTOF, ABSCIEX) (2.8 kV; data-dependent mode adquisition; MS1 scans: 350–1250 m/z for 250 ms, MS2 experiments: 100–1500 m/z for 50 ms. MS2 scans were acquired in “high sensitivity” mode). ProteinPilot default parameters were used to generate peak lists directly from 5600 TripleTof.wiff files. The Paragon algorithm^98^ of ProteinPilot (v4.5, AB SCIEX) was used to search the UniProt Mammals database (www.uniprot.org) with the following parameters: “trypsin specificity”, “cys-alkylation”, “no taxonomy restriction”, and the search effort set to “through”. The identified proteins were grouped by the Pro GroupTM algorithm. A protein group in a Pro Group Report is a set of proteins that share some physical evidence. Unlike sequence alignment analyses where full length theoretical sequences are compared, the formation of protein groups in Pro GroupTM is guided entirely by observed peptides from experimentally acquired spectra and unobserved regions of protein sequence play no role in explaining the data. Raw.txt files (ProteinSummary) containing every hit for each identified protein included information about putative hits from different species for each of the spectra that identified a protein and were probabilistically ordered. Lists were inspected and manually curated to include the first top mouse hit for each protein and human hits for proteins related to skin cells (e.g., keratins) were considered a technical contamination and removed from the analysis. Once filtered, proteins showing ProtScore > 1.3 (ProtScore = ‒log(1‒(percentage confidence/100)); 95% confidence in protein identification) were selected for further analysis. In order to be more restrictive when identifying proteins that co-immunoprecipitated with MEX3 A, a ProtScore >1.0 (90% confidence) was used in wild-type control samples to include more proteins in the background reference. UniProt IDs were used to retrieve associated gene names in the Retrieve/ID mapping tool of UniProt (https://www.uniprot.org/uploadlists/). Finally, identified proteins in the FLAG-tagged samples were compared to the ones in the wild-type background. Only proteins that were present in all FLAG-tagged biological replicates but not in wild-type were considered positive co-immunoprecipitation. MEX3A co-immunoprecipitated proteins were input in the STRING tool (https://string-db.org/) to determine putative protein-protein interactions and networks.

### Statistics and reproducibility

All statistical tests were performed using the GraphPad Prism Software (v9.4) and Microsoft Excel (v16.0). Data were analyzed for normality using the Shapiro-Wilk normality test. Analyses of significant differences between means for variables displaying normal distribution were carried out using the unpaired or paired two-tailed Student t-test for two variables or one-way ANOVA with Tukey post-hoc test for more than two groups. Similarly, variables that did not follow a normal distribution were analyzed with Mann–Whitney U test. When comparisons were carried out with relative values (normalized values and percentages), data were first normalized by using a log or arcsin transformation, respectively. *p*-values lower than 0.05 were considered as statistically different and referred as ^*^p < 0.05, ^**^p < 0.01 and ^***^p < 0.001. Data are presented as the mean ± standard error of the mean (SEM). The number of experiments carried out with biologically independent cultures/animals (*n*) is either shown as dots in the graphs or listed in the Figure Legends. When representative images are displayed without an associated quantification graph, immunostainings were performed in at least three series (4-5 sections) obtained from independent mice of the indicated genotypes.

### Reporting summary

Further information on research design is available in the Nature Portfolio Reporting Summary linked to this article.

## Supplementary information

Supplementary Information

Description of Additional Supplementary Files

Supplementary Data 1

Supplementary Data 2

Reporting Summary

## Data Availability

All materials, data, code, and associated protocols can be made available to readers promptly and without undue qualifications. All data generated in this study, including the raw files and quantitative data matrix of proteomes and transcriptomes, have been deposited online for free access by readers. RIP-seq data have been deposited in GEO under accession code GSE184404^99^. The mass spectrometry proteomics data have been deposited to the ProteomeXchange Consortium via the PRIDE^100^ partner repository with the dataset identifier PXD038983. Source data are provided with this paper Source data are provided with this paper.

## Source data

Source Data
