# Supplementary material for: Post-transcriptional control of a stemness signature by RNA-binding protein MEX3A regulates murine adult neurogenesis
Source: Nat Commun. 2023 Jan 23;14:373. doi: 10.1038/s41467-023-36054-6 (PMC9871011; doi:10.1038/s41467-023-36054-6)
Supplement: Supplementary file 1 — Supplementary Information [file 41467_2023_36054_MOESM1_ESM.pdf]

Supplementary Information

Post-transcriptional control of a stemness signature by RNA-binding protein MEX3A regulates adult neurogenesis

Ana Domingo-Muelas, Pere Duart-Abadia, Jose Manuel Morante-Redolat, Antonio Jordán-Pla, Germán Belenguer, Jaime Fabra-Beser, Lucía Paniagua-Herranz, Ana Pérez-Villalba, Adrián Álvarez-Varela, Francisco M. Barriga, Cristina Gil-Sanz, Felipe Ortega, Eduard Batlle and Isabel Fariñas

Correspondence to: isabel.farinas@uv.es, eduard.batlle@irbbarcelona.org

This PDF file includes: Supplementary Figures 1-7
Supplementary Table 1

Supplementary Figure 1

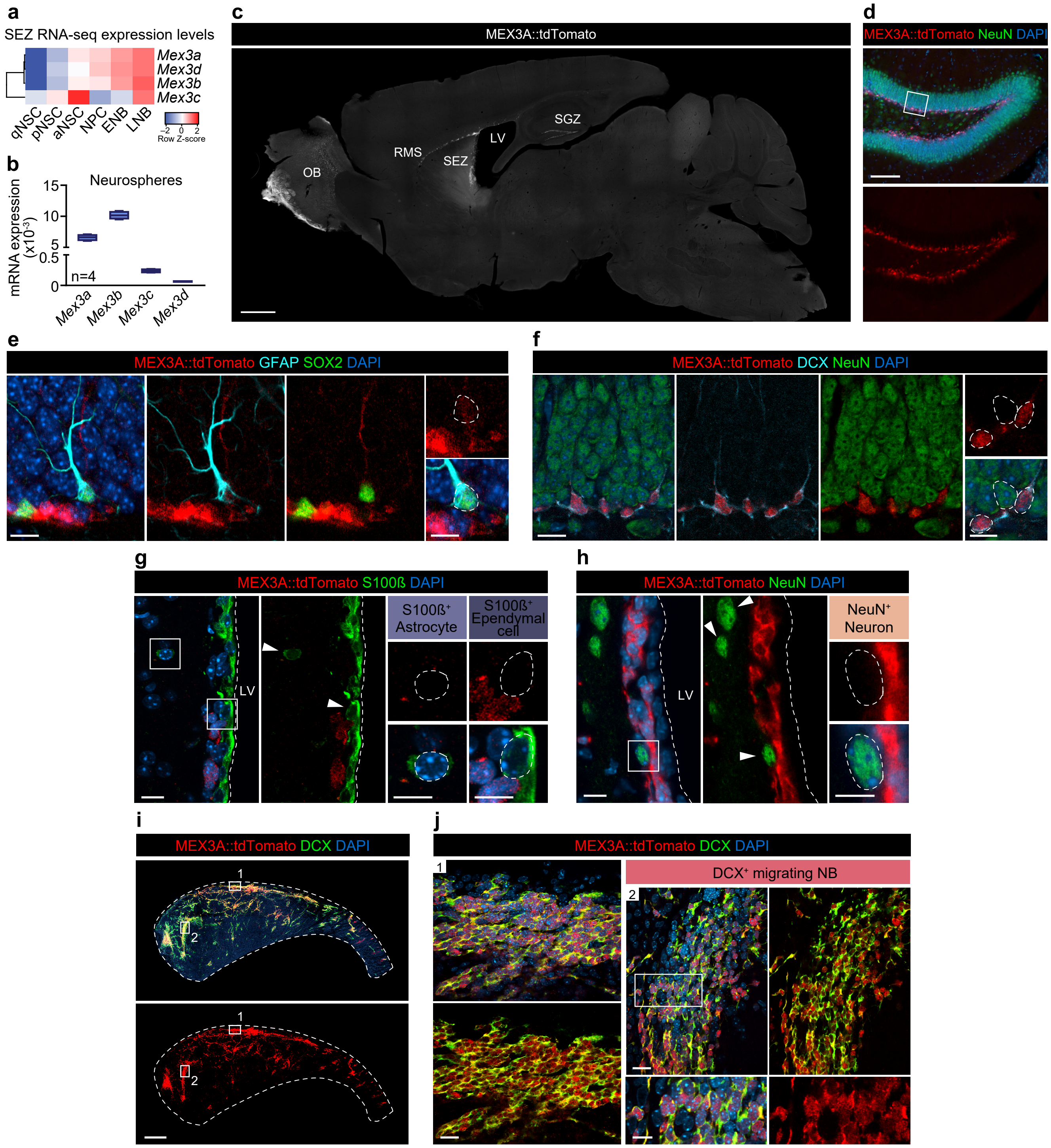

Supplementary Figure 1. *MEX3A expression in the adult mouse brain.* **a,** Heatmap showing murine *Mex3*-family members RNA-seq expression levels in SEZ sorted populations[12]. **b,** Quantification of *Mex3a, Mex3b, Mex3c* and *Mex3d* gene expression by RT-qPCR in wild-type neurosphere cultures (n=4 cultures). Data is represented as relative expression to an endogenous gene control. **c,** Panoramic brain sagittal section immunostained for MEX3A::tdTomato (white) showing the olfactory bulb (OB), the rostral migratory stream (RMS), the subependymal zone (SEZ) and the subgranular zone (SGZ). **d,** Panoramic view of the dentate gyrus (DG) of the *Mex3a*$^{+/KI}$ hippocampus. Immunohistochemistry showing expression of MEX3A::tdTomato (red) and NeuN^{+} (green) mature neurons. **e,** Immunohistochemistry showing expression of MEX3A::tdTomato (red), GFAP^{+} (cyan)/SOX2^{+} (green) NSCs in the SGZ. **f,** Immunohistochemistry showing expression of MEX3A::tdTomato (red), DCX^{+} (cyan) NBs and NeuN^{+} (green) neurons in the SGZ. **g,** Immunohistochemistry for MEX3A::tdTomato (red) and S100ß$^{+}$ (green) astrocytes in the adult SEZ. **h,** Immunohistochemistry showing expression of MEX3A::tdTomato (red) and NeuN^{+} (green) neurons in the SEZ. **i,** Panoramic en-face image of a wholemount preparation of the *Mex3a*$^{+/KI}$ SEZ. Immunohistochemistry showing expression of MEX3A::tdTomato (red) and DCX^{+} (green) NBs. **j,** High-magnification images of migrating chains of DCX^{+} neuroblasts in the dorsal (1) and anterior (2) regions of the lateral wall. DAPI (blue) was used to counterstain nuclei. White boxes localize high-magnification inserts. Dashed lines mark the lateral ventricle (LV) and contour the nuclei of cells of interest in the inserts. Box plots show median ± interquartile range and whiskers define minimum to maximum. Source data are provided as a Source Data file. Scale bars: a, 1 mm; b, 100 µm; c-h and j, 15 µm; i, 500 µm; inserts, 10 µm.

Supplementary Figure 2

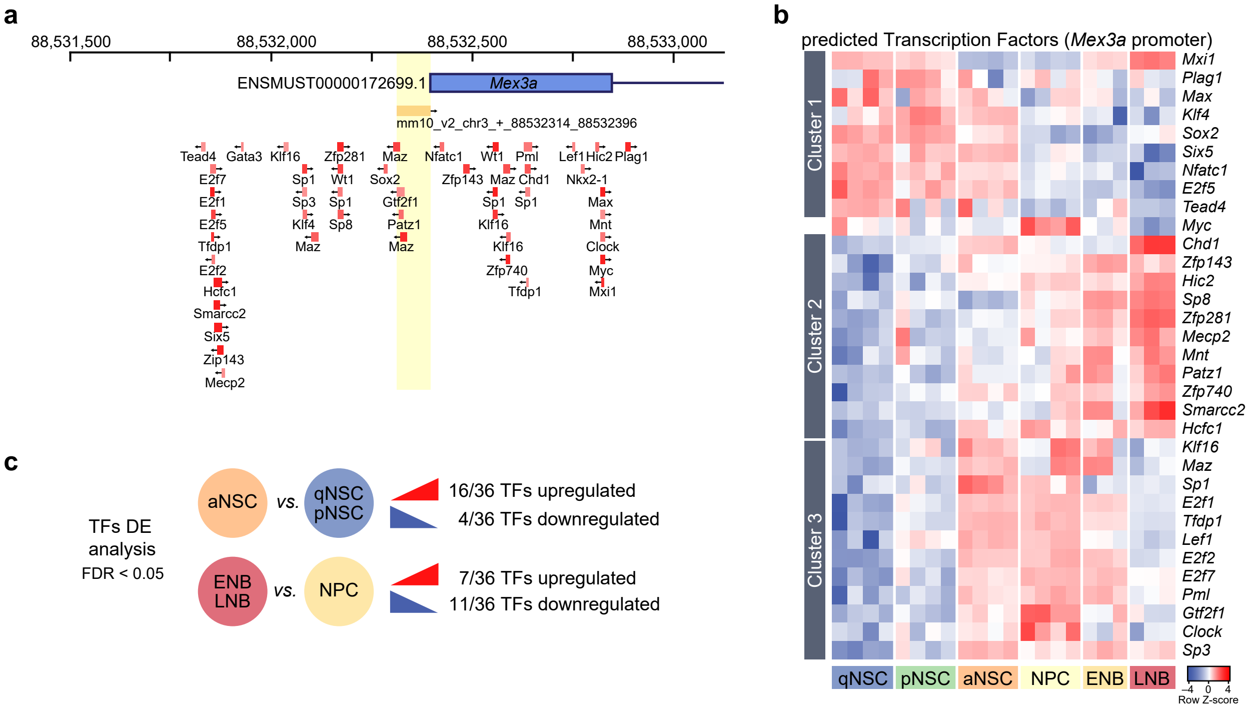

Supplementary Figure 2. *In silico* analysis of *Mex3a* promoter. a, Predicted transcription factors (TFs) with binding sites in the promoter of *Mex3a* by SwissRegulon (http://swissregulon.unibas.ch/sr/). **b,** Heatmap showing RNAseq expression pattern of these predicted transcription factors along the SEZ neurogenic lineage. **c,** Differential expression (DE) analysis of predicted transcription factors in activated *vs.* quiescent NSC and neuroblasts *vs.* NPC (FDR < 0.05).

Supplementary Figure 3

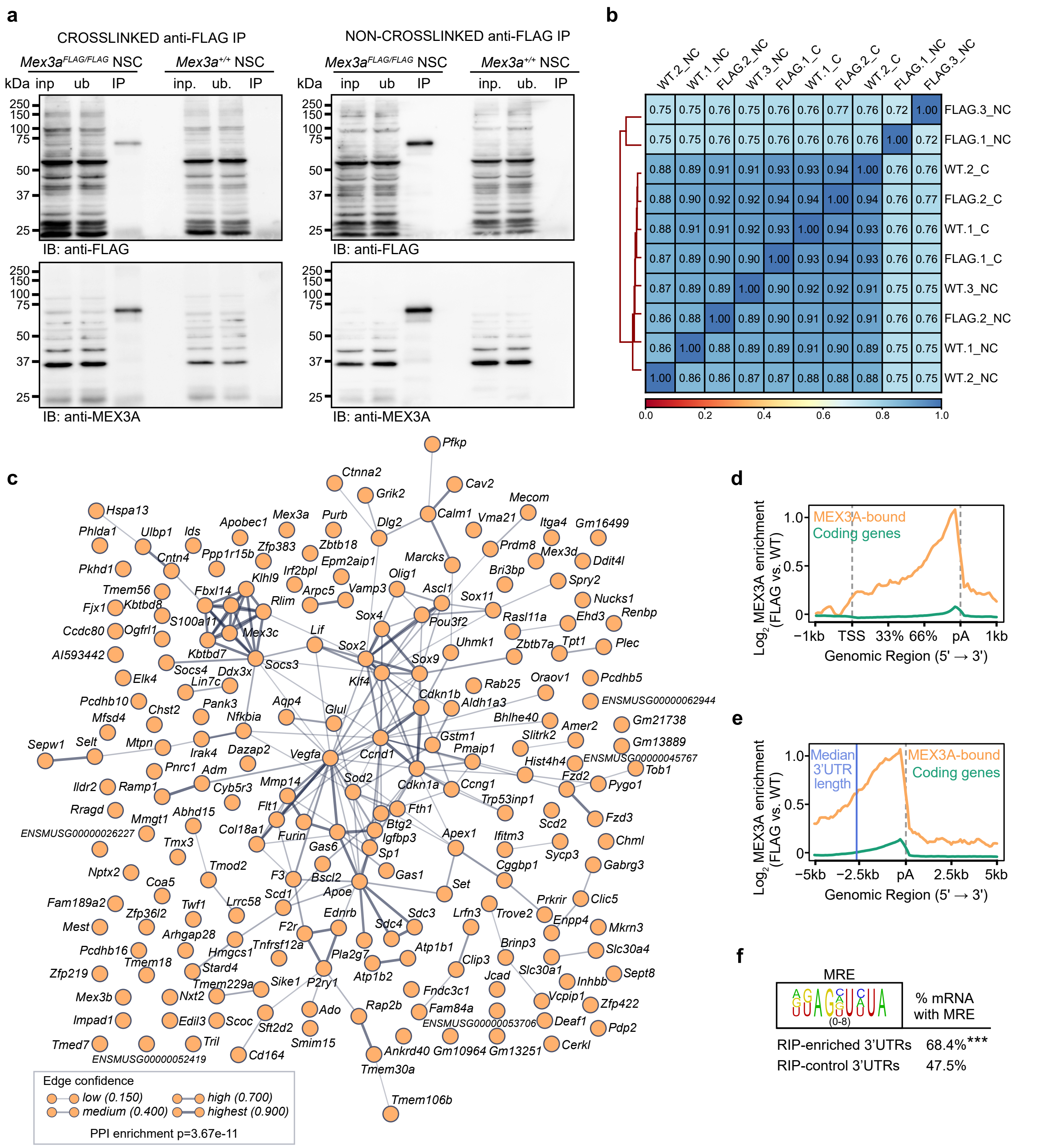

Supplementary Figure 3. *RIP-seq of MEX3A-bound RNAs in neural stem cells.* a, Western blot images for FLAG (top panels) and MEX3A (bottom panels) immunodetection after MEX3A-FLAG crosslinked (left) and native (non-crosslinked, right) immunoprecipitation (IP) in MEX3A FLAG-tagged (*Mex3a*$^{FLAG/FLAG}$) and wild-type (*Mex3a*$^{+/+}$) NSC cultures. **b,** Hierarchically clustered heatmap of pairwise Spearman correlation coefficients based on the read coverage within genomic regions. **c,** Full protein-protein interaction network generated from MEX3A-bound coding RNAs (related to **Fig. 3b**) (STRING PPI enrichment p-value=3.67x10^{-11}). Line thickness indicates the strength and confidence of the connection (https://string-db.org/). **d,** Metagene analysis of the RIP-seq signal over MEX3A-bound genes (orange) compared to the average of all protein-coding genes in the genome (green). Data is represented as log2 of the fold-change of FLAG samples (FLAG) relative to wild-type controls (WT). Genomic distance is shown as a percentage relative to TSS and pA site. **e,** Protein-coding metagene analysis of sequence enrichment after MEX3A IP showing a 5 kb upstream and downstream window from the pA. The median 3'UTR length for MEX3A-bound RNAs (blue) is also included. **f,** Schematic representation of the MEX-3 recognition element (MRE) consensus sequence from C. elegans[45]. Estimation of the percentage of RIP-enriched transcripts with at least one MRE in their annotated 3'UTRs. Enrichment was calculated by comparing 3'UTRs from non-IP mRNAs as controls (p-value<0.0001). inp: input; ub: unbound fraction; IP: immunoprecipitated fraction; IB: immunoblot; TSS: transcription start site; pA: poly-A site.

Supplementary Figure 4

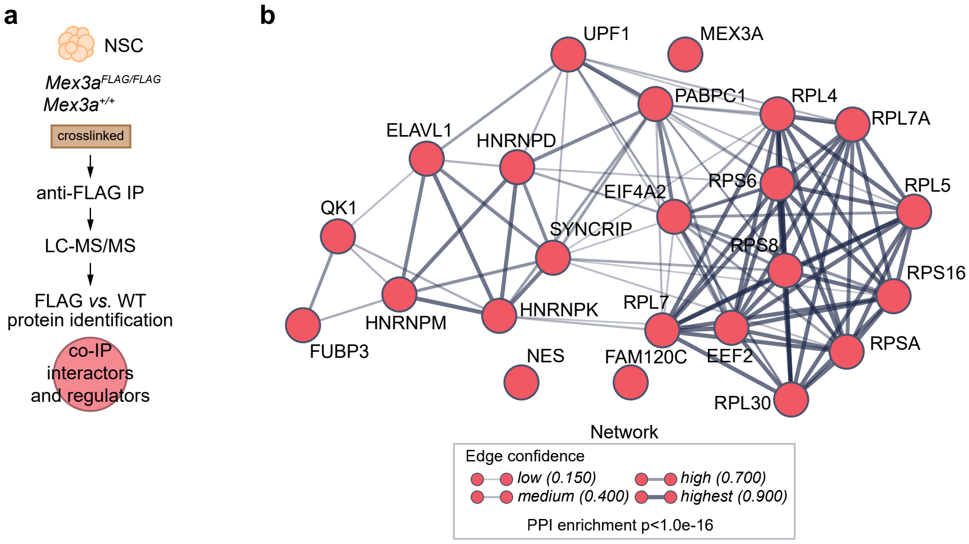

Supplementary Figure 4. ***Potential interactors and co-regulators of MEX3A.*** **a,** Schematic representation of MEX3A protein co-immunoprecipitation (co-IP). IP: immunoprecipitation; LC-MS/MS: liquid chromatography coupled to tandem mass spectrometry. **b,** STRING network generated from MEX3A co-immunoprecipitated proteins (STRING PPI enrichment p-value<1.0×10^{-16}). Line thickness indicates the strength and confidence of the connection (https://string-db.org/).

Supplementary Figure 5

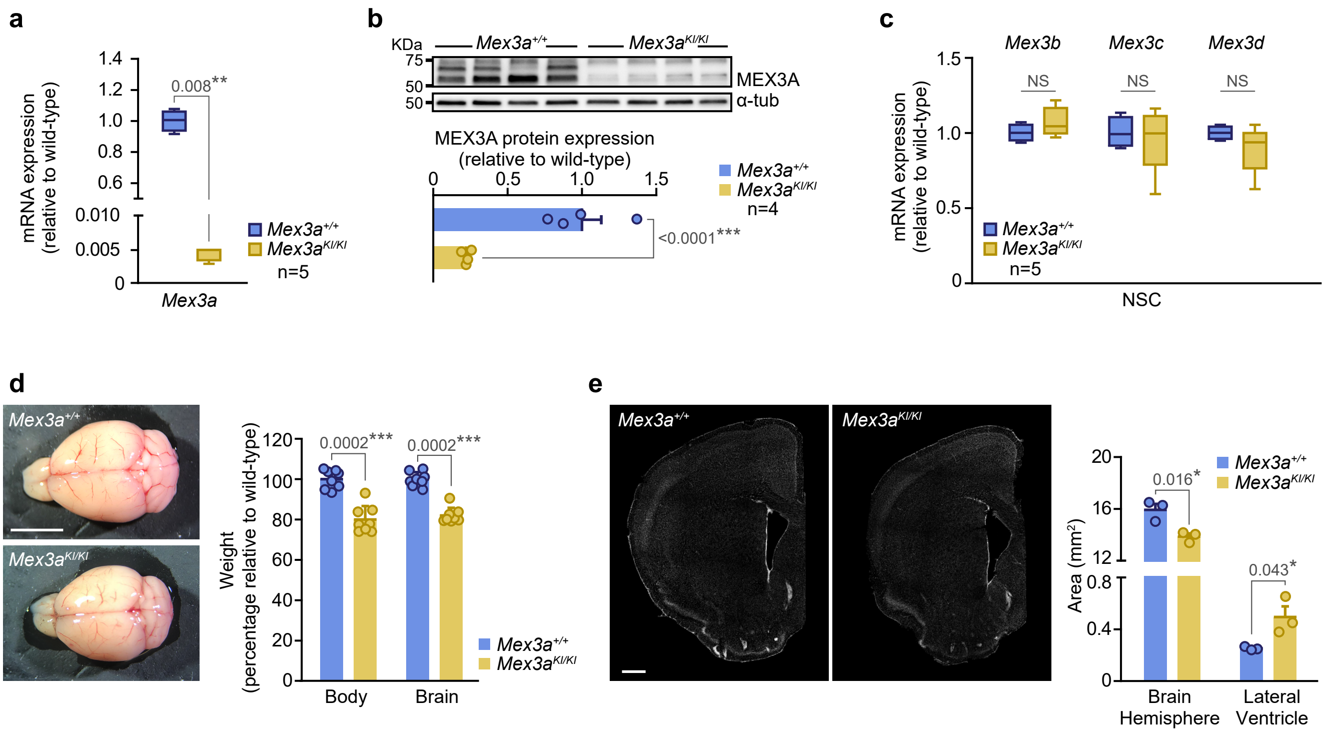

Supplementary Figure 5. ***Mex3a KI mice as a model to study MEX3A-deficiency.*** **a,** Quantification of *Mex3a* gene expression by RT-qPCR in wild-type (*Mex3a*$^{+/+}$) and KI homozygous (*Mex3a*$^{KI/KI}$) NSC cultures (**p-value<0.01, n=4 wild-type and 5 KI/KI cultures, by two-tailed Mann-Whitney U). **b,** Immunoblot images for MEX3A and α-TUBULIN in *Mex3a*$^{+/+}$ and *Mex3a*$^{KI/KI}$ NSC cultures (top panel). Quantification of MEX3A protein levels by Western blot in *Mex3a*$^{+/+}$ and *Mex3a*$^{KI/KI}$ NSC cultures (bottom panel) (***p-value<0.001, n=4 cultures, by unpaired two-tailed Student's t-test). **c,** RT-qPCR expression data for *Mex3b*, *Mex3c* and *Mex3d* in wild-type and MEX3A-deficient neurosphere cultures (*Mex3b* NS=0.221, *Mex3c* NS=0.651, *Mex3d* NS=0.252, n=4 wild-type and 5 KI/KI cultures, by unpaired two-tailed Student's t-test). Data is represented as fold change relative to wild-type for each gene. **d,** Image showing the size and physical appearance of *Mex3a*$^{+/+}$ and *Mex3a*$^{KI/KI}$ adult brains (left panel). Body and brain weight from wild-type and homozygous adult mice (right panel) (***p-value<0.001, n=8 mice, by unpaired two-tailed Mann-Whitney U). Data is represented as a percentage relative to wild-type. **e,** Representative DAPI-stained images of coronal sections from *Mex3a*$^{+/+}$ and *Mex3a*$^{KI/KI}$ brains (left panel). Quantification of the mean hemisphere area and mean ventricular lumen area for *Mex3a*$^{+/+}$ and *Mex3a*$^{KI/KI}$ brain coronal sections (right panel) (*p-value<0.05, n=3 mice, by unpaired two-tailed Student's t-test). Graphs represent mean values and all error bars show SEM. Box plots show median ± interquartile range and whiskers define minimum to maximum. Exact p-values and the number of biologically independent samples (represented as dots) used are indicated in the graphs. Source data are provided as a Source Data file. Scale bars: d, 500 mm; e, 500 µm.

Supplementary Figure 6

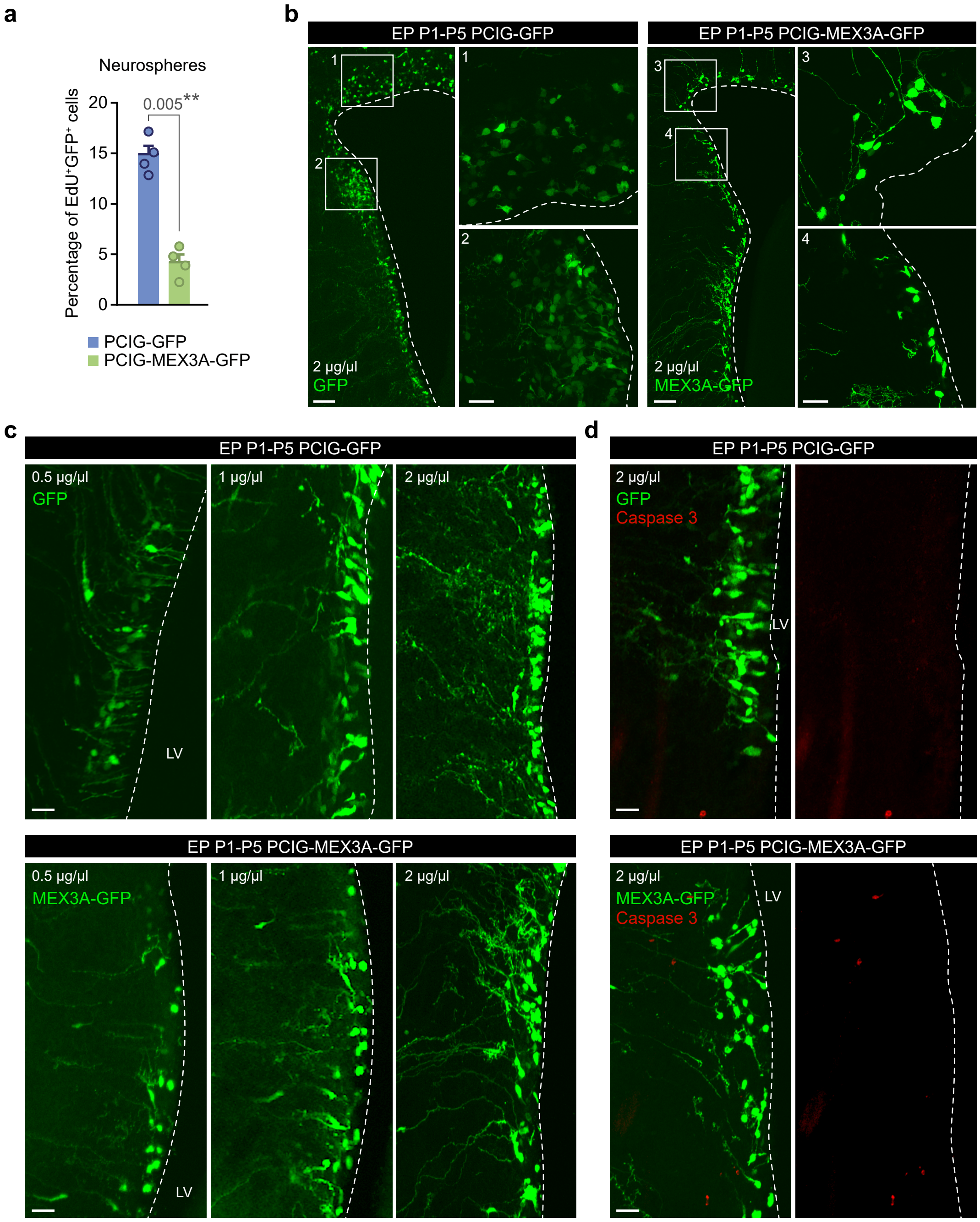

Supplementary Figure 6. *Overexpression of MEX3A in neurospheres and in the subependymal zone.* **a**, Quantification of the percentage of EdU⁺GFP⁺ cells 24h after overexpression of 4.5 µg of PCIG-GFP or PCIG-MEX3A-GFP in neurosphere wild-type cultures (**p-value<0.01, n=4 cultures, by paired two-tailed Student's t-test). **b**, GFP and MEX3A-GFP signal (green) in the lateral ventricle (LV) of P5 mice 4 days after postnatal day 1 (P1) electroporation (EP) with PCIG-GFP (left) and PCIG-MEX3A-GFP (right) plasmids. Inserts highlight the morphology of electroporated cells in both conditions. Note that while cells divide and dilute the plasmid in the empty vector condition, cells overexpressing MEX3A show a quiescent-like NSC morphology and retain high levels of the GFP-expressing plasmid. This evidences cell cycle arrest. **c**, GFP and MEX3A-GFP signal (green) after electroporation with different concentrations (0.5 µg/µl, 1 µg/µl and 2 µg/µl) of PCIG-GFP (top) and PCIG-MEX3A-GFP (bottom) episomal plasmids. **d,** Immunostaining for cleaved Caspase-3 in these conditions. Graphs represent mean values and all error bars show SEM. Exact p-values and the number of biologically independent samples (represented as dots) used are indicated in the graphs. Source data are provided as a Source Data file. Scale bars: b, 100 µm (inserts, 50 µm); c-d, 50 µm.

Supplementary Figure 7

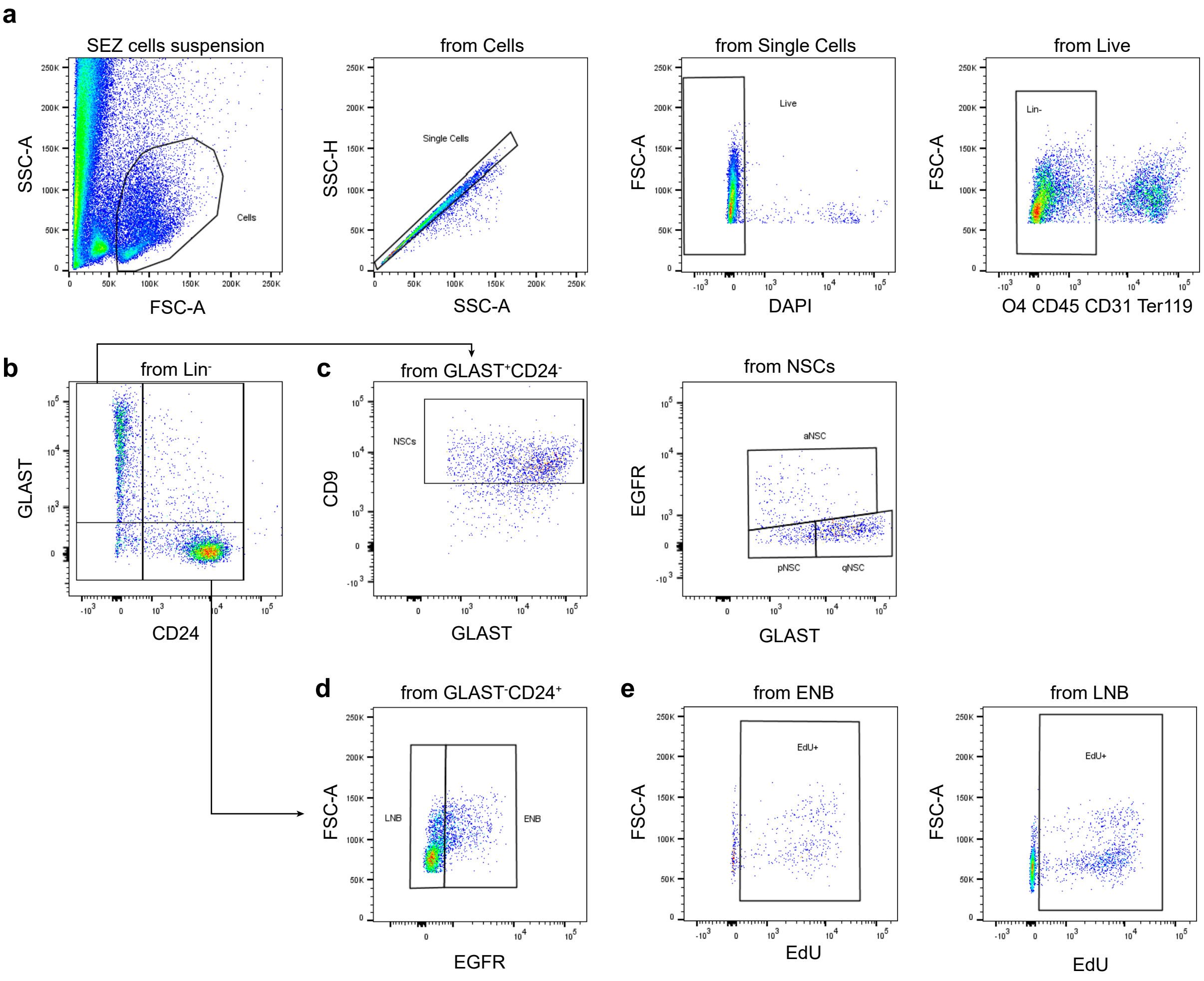

Supplementary Figure 7. *FACS gating strategy for SEZ populations.* a, Gating strategy used to identify SEZ lineage-negative (Lin$^-$) live cells after excluding oligodendrocytes (O4$^+$), microglia (CD45$^+$), endothelial cells (CD31$^+$) and erythrocytes (Ter119$^+$). **b,** GLAST and CD24 plot showing gating to identify NSCs (**c**) and NBs (**d**). Both (**a**) and (**b**) are used as a first step for every SEZ FACS experiment (**Fig. 2f,g**, **Fig. 4c,g** and **Fig. 6c,d**). **c,** Gating strategy to identify NSC populations. This is used in **Fig. 2f,g** and **Fig. 4c,g**. **d** and **e,** Gating strategy to identify NBs (**d**) used in **Fig. 2f** and **Fig. 6c,d**, and to quantify EdU$^+$ cells in ENB and LNB (**e**) in **Fig. 6d**.

Supplementary Table 1

Gene	Use	Sequence (5’–3’)
Mex3a_3XFlag-F	Genotyping	ACTGCATGGTGTGCTTTGAG
Mex3a_3XFlag-R	Genotyping	CAAGCACCTTGCCCTGTAGT
Mex3a_KI-F	Genotyping	GGCTTAGGGGTGGGTCTATC
Mex3a_WT-R	Genotyping	GATCAAGAGCCAGCTGAAGG
Mex3a_KI-R	Genotyping	GCTTCTTGTAATCGGGGATG
Guide cRNA 19	CRISPR/Mouse generation	UUUCCUAAGCUCCAUGUCCCGUUUUAGAGCUAUGCUGUUUUG
ssDNA 19t	CRISPR/Mouse generation	TGCCCTGTAGTCACTGATTTTTCAAAAAGCCCTACAGGTCCAGG GCAGCACCCGAGGGAGCGGGCCCAGGAGACCAGGGACATGG AGCTTACTTGTCGTCGTCGTCCTTGTAGTCGATGTCGTGGTCCT TGTAGTCACCGTCGTGGTCCTTGTAGTCGGAAAATATTCGGAT GGCTTGTGTGGCGGTGATGTG
Guide cRNA 26	CRISPR/Mouse generation	AGACCAGGGACAUGGAGCUUGUUUUAGAGCUAUGCUGUUUUG
ssDNA 26t	CRISPR/Mouse generation	CATGGAGTGTGCAGTACGCATCTGCGAGAGGACGGACCCAGA GTGTCCTGTCTGCCACATCACCGCCACACAAGCCATCCGAATA TTTTCCGACTACAAGGACCACGACGGTGACTACAAGGACCACG ACATCGACTACAAGGACGACGACGACAAGTAAGCTCCATGTCC CTGGTCTCCTGGGCCCGCTCCC
Mex3a_x1–F	Expression analysis	GAGCTGCGACTGAAGGG
Mex3a_x2–R	Expression analysis	CTCAGAGCCTTAATCTTGCAG
Mex3b_x1–F	Expression analysis	GTCGCGGAGATCGTAGGG
Mex3b_x2–R	Expression analysis	ACATCCTCCTTCCTGCCC
Mex3c_x1–F	Expression analysis	GCATGTCGCTGAGATTGTC
Mex3c_x2–R	Expression analysis	TCTTCTTTCCGTCCAGTG
Mex3d_x1–F	Expression analysis	CAGTTGAACGTGATCGG
Mex3d_x2–R	Expression analysis	GTATTGGTCTTGGCACG
Gapdh_x2–F	Expression analysis	GCCGGTGCTGAGTATGTCG
Gapdh_x3–R	Expression analysis	AGAAGGGGCGGAGATGATG
Sdc4_x2–F	Expression analysis	TCGGATGACTTTGAGCTCTCGG
Sdc4_x3–R	Expression analysis	CAAGGGCTCAATCACTTCAGGG
Aqp4_x2–F	Expression analysis	AGTGTGGTTGGAGGATTG
Aqp4_x3–R	Expression analysis	AATAGTGAACACCAACTGG

Supplementary Table 1. Sequences of oligonucleotides (primers for genotyping and expression analysis, CRISPR guides).
