# Supplementary material for: Post-transcriptional control of a stemness signature by RNA-binding protein MEX3A regulates murine adult neurogenesis
Source: Nat Commun. 2023 Jan 23;14:373. doi: 10.1038/s41467-023-36054-6 (PMC9871011; doi:10.1038/s41467-023-36054-6)
Supplement: Supplementary file 2 — Description of Additional Supplementary Files [file 41467_2023_36054_MOESM2_ESM.pdf]

Description of Additional Supplementary Files

File name: Supplementary Data 1

Description: MEX3A RIP-seq results. Table includes all MEX3A identified targets, RIP enrichment and statistics, biotype information and MEX-3 recognition element (MRE) presence.

File name: Supplementary Data 2

Description: Gene Ontology enrichment analysis of MEX3A regulon. STRING Biological Process ontology analysis of MEX3A identified targets. In bold, most relevant terms in NSCs that have been included in Fig. 3c.
