# Supplementary material for: Post-transcriptional control of a stemness signature by RNA-binding protein MEX3A regulates murine adult neurogenesis
Source: Nat Commun. 2023 Jan 23;14:373. doi: 10.1038/s41467-023-36054-6 (PMC9871011; doi:10.1038/s41467-023-36054-6)
Supplement: Supplementary file 5 — Reporting Summary [file 41467_2023_36054_MOESM5_ESM.pdf]

nature portfolio

Corresponding author(s): Isabel Fariñas

Last updated by author(s): Dec 22, 2022

Reporting Summary

Nature Portfolio wishes to improve the reproducibility of the work that we publish. This form provides structure for consistency and transparency in reporting. For further information on Nature Portfolio policies, see our Editorial Policies and the Editorial Policy Checklist.

Statistics

For all statistical analyses, confirm that the following items are present in the figure legend, table legend, main text, or Methods section.

n/a	Confirmed	
☐	☒	The exact sample size (*n*) for each experimental group/condition, given as a discrete number and unit of measurement
☐	☒	A statement on whether measurements were taken from distinct samples or whether the same sample was measured repeatedly
☐	☒	The statistical test(s) used AND whether they are one- or two-sided<br>*Only common tests should be described solely by name; describe more complex techniques in the Methods section.*
☒	☐	A description of all covariates tested
☐	☒	A description of any assumptions or corrections, such as tests of normality and adjustment for multiple comparisons
☐	☒	A full description of the statistical parameters including central tendency (e.g. means) or other basic estimates (e.g. regression coefficient) AND variation (e.g. standard deviation) or associated estimates of uncertainty (e.g. confidence intervals)
☐	☒	For null hypothesis testing, the test statistic (e.g. *F*, *t*, *r*) with confidence intervals, effect sizes, degrees of freedom and *P* value noted<br>*Give P values as exact values whenever suitable.*
☒	☐	For Bayesian analysis, information on the choice of priors and Markov chain Monte Carlo settings
☒	☐	For hierarchical and complex designs, identification of the appropriate level for tests and full reporting of outcomes
☒	☐	Estimates of effect sizes (e.g. Cohen's *d*, Pearson's *r*), indicating how they were calculated

Our web collection on statistics for biologists contains articles on many of the points above.

Software and code

Policy information about availability of computer code

Data collection	Microphotographs were collected with Olympus FV10i-SW (version 2.1) for confocal images or Nikon NIS-Elements BR (v5.11) for widefield images. Flow Citometry data were collected with BD FACSDiva (version 8.0.2).
Data analysis	All image analyses were performed using NIH ImageJ-Win64 Fiji (version 1.53f) or Olympus FV10-ASW (version 04.02). Flow cytometry analysis was performed in FlowJo (version 11). Different steps of the bioinfirnatics analyses required the use of the following software/packages: FastQC (version 0.11.7), Cutadapt (version 1.16), Trimmomatic (version 0.36), HISAT2 (version 2.1.0), deepTools2 Python package (version 3.1.1), R package Rsubread (version 1.6.2), DESeq2 (version 1.16.1), ComplexHeatmap (version 1.10.2), ggplot2 (version 3.0.0), R and Python software ngs.plot (version 2.61), MEME Suite (version 4.11.4) and ProteinPilot (version 4.5) . Statistical analyses were performed using GraphPad Prism (Version 9.4) and Microsoft Excel (Version 16.0). Images and data were processed for visualization with Adobe Photoshop (version 23.1) and Adobe Illustrator (version 26.0).

For manuscripts utilizing custom algorithms or software that are central to the research but not yet described in published literature, software must be made available to editors and reviewers. We strongly encourage code deposition in a community repository (e.g. GitHub). See the Nature Portfolio guidelines for submitting code & software for further information.

Data

Policy information about availability of data

All manuscripts must include a data availability statement. This statement should provide the following information, where applicable:

- Accession codes, unique identifiers, or web links for publicly available datasets
- A description of any restrictions on data availability
- For clinical datasets or third party data, please ensure that the statement adheres to our policy

RIP-seq data have been deposited in GEO under accession code GSE184404 [https://www.ncbi.nlm.nih.gov/geo/query/acc.cgi?acc=GSE184404]. The mass spectrometry proteomics data have been deposited to the ProteomeXchange Consortium via the PRIDE partner repository with the dataset identifier PXD038983. As reference datasets we used the Mus musculus GRCm38 (mm10) genome assembly (https://www.ncbi.nlm.nih.gov/assembly/GCF_000001635.20/) and mouse genome annotation version M16 (Ensembl 91) (https://www.gencodegenes.org/mouse/release_M16.html). Source data are provided with this paper.

Human research participants

Policy information about studies involving human research participants and Sex and Gender in Research.

Reporting on sex and gender	No human research participants were used in this study.
Population characteristics	No human research participants were used in this study.
Recruitment	No human research participants were used in this study.
Ethics oversight	No human research participants were used in this study.

Note that full information on the approval of the study protocol must also be provided in the manuscript.

Field-specific reporting

Please select the one below that is the best fit for your research. If you are not sure, read the appropriate sections before making your selection.

☒ Life sciences ☐ Behavioural & social sciences ☐ Ecological, evolutionary & environmental sciences

For a reference copy of the document with all sections, see nature.com/documents/nr-reporting-summary-flat.pdf

Life sciences study design

All studies must disclose on these points even when the disclosure is negative.

Sample size	No statistical method was used to predetermine sample size. Sample sizes were based on previously published experiments by us and others (see as examples: Porlan et al. Nat Cell Biol., 2014; Llorens-Bobadilla et al., Cell Stem Cell, 2015; Kalamakis et al., Cell, 2019; Delgado et al. Science, 2021, Belenguer et al., Cell Stem Cell, 2021) and on our own un-published data.
Data exclusions	All experimental data were included in the manuscript. In those few cases were samples did not reach the expected quality standard (due to technical problems during processing or manipulation), they were discarded before data acquisition, to avoid bias. In the mouse proteomics experiments, human hits for proteins related to skin cells (e.g., keratins) were considered a technical contamination and removed from the analysis.
Replication	All experiments were performed at least twice, in different days, with different samples (when possible) and two or more researchers were involved in both experimental procedure and data acquisition and analysis. Final data was obtained including all independent biological replicates. All replication attemps were successful.
Randomization	In this type of study, experimental groups are highly dependent on the limiting offspring of the required genotypes. In each case, available littermates were allocated in the experimental groups according to their age and genotype ensuring equal distribution of males and females in the experiment. No other covariates were considered. In case of treatments, such as BrdU/EdU pulses, all groups received the same doses, so there was no need for randomization.
Blinding	The investigators were not blinded to allocation during experiments and outcome assessment because of the chosen experimental design: in all cases where different genotypes were compared, analysis settings were established for the control group and then kept unvariably to the rest of the groups.

Reporting for specific materials, systems and methods

We require information from authors about some types of materials, experimental systems and methods used in many studies. Here, indicate whether each material, system or method listed is relevant to your study. If you are not sure if a list item applies to your research, read the appropriate section before selecting a response.

Materials & experimental systems

n/a	Involved in the study
☐	☒ Antibodies
☒	☐ Eukaryotic cell lines
☒	☐ Palaeontology and archaeology
☐	☒ Animals and other organisms
☒	☐ Clinical data
☒	☐ Dual use research of concern

Methods

n/a	Involved in the study
☒	☐ ChIP-seq
☐	☒ Flow cytometry
☒	☐ MRI-based neuroimaging

Antibodies

Antibodies used

Primary antibodies:
rabbit anti-AQP4 (Sigma, cat no. HPA014784, 1:200)
rabbit anti-DsRed (Takara, cat. no. 632496, 1:400)
chicken anti-GFP (Aveslabs, cat. no. GFP-1020, 1:500)
chicken anti-GFAP (Millipore, cat. no. AB5541, 1:800)
mouse anti-ASCL1 (BD, cat. no. 556604, clone 24B72D11.1, 1:100)
chicken anti-DCX (Abcam, cat. no. ab153668, 1:500)
rabbit anti-DCX (Abcam, cat. no. ab18723, 1:1000)
goat anti-DCX (Santa Cruz, cat. no. sc-8066, 1:300)
mouse anti-NeuN (Millipore, cat. no. MAB377, clone A60, 1:200)
goat anti-POU3F2/BRN2 (Santa Cruz Biotechnology; cat no. sc-6029, 1:50)
mouse anti-S100b (Sigma, cat. no. S2532, clone SH-B1, 1:500)
rabbit anti-Ki67 (Abcam, cat. no. ab15580, 1:300)
goat anti-SOX2 (R&D, cat. no. AF2019, 1:600)
rabbit anti-SOX9 (Abcam, cat. no. ab184547, 1:1000)
rabbit anti-bIII-TUBULIN (Sigma, cat. no. T2200, 1:300)
rat anti-BrdU (Abcam, cat. no. ab6326, 1:800)
goat anti-FLAG (Abcam, cat. no. ab1257, 1:1000)
mouse anti-FLAG antibody (Sigma, cat. no. F1804, clone M2, 10 µg per IP)
anti-CD45-BV421 (BD Bioscience, cat. no. 563890, 1:200)
anti-TER119-BV421 (BD Bioscience, cat. no. 563998, 1:200)
anti-CD31-BV421 (BD Bioscience, cat. no. 563356, 1:100)
anti-O4-biotin (Miltenyi, cat. no. 130-095-895, 1:30)
anti-CD24-PerCP-Cy5.5 (BD Bioscience, cat. no. 562360, 1:300)
anti-CD24-BB700 (BD Bioscience, cat. no. 746122, 1:300)
anti-CD9-APC-VIO770 (Miltenyi, cat. no. 130-102-384, 1:20)
anti-GLAST-APC (Miltenyi, cat. no. 130-095-814, 1:20)
anti-SDC4–APC (Miltenyi, cat. no. 130-109-831, clone REA640, 1:200)

Secondary antibodies:
Alexa Fluor® 488 Donkey Anti-chicken (Jackson ImmunoResearch, 703-545-155, 1:800)
Alexa Fluor® 488 Donkey Anti-mouse (Molecular Probes, cat. no. A21202, 1:800)
Alexa Fluor® 488 Donkey Anti-rabbit (Jackson ImmunoResearch, cat. no. 711-547-003, 1:800)
Alexa Fluor® 647 Donkey Anti-chicken (Jackson ImmunoResearch, cat. no. 703-606-155, 1:800)
Alexa Fluor® 647 Donkey Anti-goat (Molecular Probes, cat. no. A21447, 1:800)
Cy3 Donkey anti-chicken (Jackson ImmunoResearch, cat. no. 703-165-155, 1:800)
Cy3 Donkey anti-mouse (Jackson ImmunoResearch, cat. no. 715-165-151, 1:800)
Cy3 Donkey anti-rabbit (Jackson ImmunoResearch, cat. no. 711-165-152, 1:800)
Cy3 Donkey anti-rat (Jackson ImmunoResearch, cat. no. 712-165-153, 1:800)
BV421 streptavidin (BD Bioscience, cat. no. 563259, 1:300)
EGF-488 (Molecular Probes, cat. no. E13345, 1:300)

Validation

Aquaporin-4 (AQP4): Montgomery et al, Cell Rep 31(2):107500, 2020; Liddelow et al, Nature 541(7638):481-487, 2017.
DsRed/tdTomato: Kelley et al, Neuron 98(2):306-319.e7, 2018; Zhou et al, Nat Neurosci 21(3):440-446, 2018.
Glial Fibrillary Acidic Protein (GFAP): Delgado et al, Neuron 83(3):572-85, 2014; Porlan et al, Nat Cell Biol 16(7):629-38, 2014; Marques-Torrejon et al, Cell Stem Cell 12(1):88-100, 2013; Ferron et al, Cell Stem Cell 7(3):367-79, 2010.
ASCL1/MASH1 (achaete-scute family bHLH transcription factor 1): Blomfield et al, Elife 8:e48561, 2019; Park et al, Cell Stem Cell 21(3):411, 2017; Sommer et al, Neuron 15(6):1245-58, 1995.
Doublecortin (DCX): ab153668 (Kuo et al, Life Sci 254:117755, 2020); ab18723 (Bott et al, J Neurosci 40(19):3720-3740, 2020; Wu et al, Nat Commun 7:10533, 2016; Wang et al, Nat Commun 7:10481, 2016); sc-8066 (Urban et al, Science 353(6296):292-5, 2016; Ferron et al, Nature 475(7356):381-5, 2011; Snyder, J Neurosci 29(46):14484-95, 2009). 28(2):285-299.e9
NeuN (neuronal nuclear protein): Ahrens et al, Nat Neurosci 18(1):104-11, 2015; DeBoer et al, J Neurosci 34(10):3674-86, 2014.
POU3F2/BRN2 (POU class 3 homeobox 2): Inoue et al, Mol Cell Biol 32(13):2618-27, 2012; Pulvers et al, Development 136(11):1859-68, 2009.
S100b (S100 calcium binding protein B): Montalban-Loro et al, Nat Commun 10(1):1726, 2019; Marques-Torrejon et al, Cell Stem Cell 12(1):88-100.

Ki67: Delgado et al, Neuron 83(3):572-85, 2014; Ferron et al, Nature 475(7356):381-5, 2011.
SOX2 (SRY-box transcription factor 2): Skelly et al, Cell Stem Cell 27(3):459-469.e8, 2020; Perez-Villalba et al, J Neurosci 38(4):814-825, 2018.
SOX9 (SRY-box transcription factor 9): Fabra-Beser et al, J Neurosci 41(33):6969-6986, 2021.

bIII-TUBULIN: Lopez-Fabuel et al, Nat Commun 13(1):536, 2022; Tsunemoto et al, Nature 557(7705):375-380, 2018.
BrdU: Montalban-Loro et al, Nat Commun 10(1):1726, 2019; Marques-Torrejon et al, Cell Stem Cell 12(1):88-100.

Flow cytometry antibodies and reagents (anti-CD45-BV421 (BD Bioscience, cat. no. 563890, 1:200); anti-TER119-BV421 (BD Bioscience, cat. no. 563998, 1:200); anti-CD31-BV421 (BD Bioscience, cat. no. 563356, 1:100); anti-O4-biotin (Miltenyi, cat. no. 130-095-895, 1:30); BV421 streptavidin (BD Bioscience, cat. no. 563259, 1:300); anti-CD24-PerCP-Cy5.5 (BD Bioscience, cat. no. 562360, 1:300); anti-CD24-BB700 (BD Bioscience, cat. no. 746122, 1:300); anti-CD9-APC-VIO770 (Miltenyi, cat. no. 130-102-384, 1:20); anti-GLAST-APC (Miltenyi, cat. no. 130-095-814, 1:20), EGF-488 (Molecular Probes, cat. no. E13345, 1:300)) have been previously validated by our and other labs in the adult neurogenesis field (Belenguer et al, Cell Stem Cell 28(2):285-299.e9, 2021; Belenguer et al, STAR Protoc 2(2):100425, 2021; Kalamakis, Cell 176(6):1407-1419.e14, 2019; Llorens-Bobadilla et al, Cell Stem Cell 17(3):329-40, 2015; Codega et al, Neuron 82(3):545-59, 2014).

Animals and other research organisms

Policy information about studies involving animals; ARRIVE guidelines recommended for reporting animal research, and Sex and Gender in Research

Laboratory animals

Mex3atm1(tdTomato-T2A-CreERT2)EBa reporter mice were generated at the Batlle lab as previously published (Barriga et al, Cell Stem Cell, 2017). Heterozygous knock-in (KI) mice (Mex3a+/KI) were used to study Mex3a expression, while homozygous KI mice (Mex3aKI/KI) were used as hypomorphs to study Mex3a deficiency. Littermates not carrying the KI cassette (Mex3a+/+) were used as wild-type controls.
Mex3atm2(3X-flag)EBa tagged MEX3A mice were generated by insertion of a 3X-FLAG sequence by CRISPR/Cas9 technology and first described in this study. Homozygous FLAG (Mex3aFLAG/FLAG) were used to perform RIP-seq experiments.

2-4 months-old mice were used for flow cytometry analysis, neurosphere culture, in vivo phenotypic analysis and neurogenesis experiments. 9 months old mice were used in flow cytometry phenotypic analysis. 12 months-old mice were also used for neurogenesis studies.
Postnatal day 1.5 (P1.5) wild-type (C57B6/j) pups were used for overexpression experiments by in vivo postnatal electroporation.

All mice were bred and housed, under 12 h periods of light/darkness, room temperature of 20–22 °C, 40-70% humidity and free accessible diet of pellets and water.

Wild animals

The study did not involve wild animals.

Reporting on sex

Both male and female mice were used in the study.

Field-collected samples

The study did not involve samples collected from the field.

Ethics oversight

All experiments involving mice were performed at the animal housing facility (University of Valencia, Servei Central de Suport a la Investigació Experimental – SCSIE, Burjassot) in compliance with European Union 2010/63/UE and Spanish RD-53/2013 guidelines and under official veterinary supervision. All experimental procedures were approved by the Ethics Committee of University of Valencia (CEEA: 2015/VSC/PEA/00132 and 00133).

Note that full information on the approval of the study protocol must also be provided in the manuscript.

Flow Cytometry

Plots

Confirm that:

☒ The axis labels state the marker and fluorochrome used (e.g. CD4-FITC).

☒ The axis scales are clearly visible. Include numbers along axes only for bottom left plot of group (a 'group' is an analysis of identical markers).

☒ All plots are contour plots with outliers or pseudocolor plots.

☒ A numerical value for number of cells or percentage (with statistics) is provided.

Methodology

Sample preparation

SEZ populations were analyzed as previously described (Belenguer et al, Cell Stem Cell 28(2):285-299.e9, 2021; Belenguer et al, STAR Protoc 2(2):100425, 2021). Briefly, SEZs were minced and enzymatically digested using the Neural tissue dissociation kit (T) (Miltenyi, cat no. 130-093-231) following the instructions of the manufacturer in a gentleMACS Octo Dissociator with heaters (Miltenyi). Digestion was quenched with 3 ml of 100 µg/ml trypsin inhibitor (Sigma, cat no. T6522) and the digested pieces were mechanically dissociated pipetting up and down 20-30 times through a plastic Pasteur pipette and the cell suspension was filtered through a 40 µm nylon filter. The eluted fraction was pelleted (300 xg, 10 min), resuspended in 100 µl of flow cytometry blocking buffer (0.1% Glucose, 10 mM HEPES, 2 mM EDTA and 0.5% BSA in HBSS)) and incubated with

the specific primary antibodies and reagents (anti-CD45-BV421 (BD Bioscience, cat. no. 563890, 1:200); anti-TER119-BV421 (BD Bioscience, cat. no. 563998, 1:200); anti-CD31-BV421 (BD Bioscience, cat. no. 563356, 1:100); anti-O4-biotin (Miltenyi, cat. no. 130-095-895, 1:30); BV421 streptavidin (BD Bioscience, cat. no. 563259, 1:300); anti-CD24-PerCP-Cy5.5 (BD Bioscience, cat. no. 562360, 1:300); anti-CD24-BB700 (BD Bioscience, cat. no. 746122, 1:300); anti-CD9-APC-VIO770 (Miltenyi, cat. no. 130-102-384, 1:20); anti-GLAST-APC (Miltenyi, cat. no. 130-095-814, 1:20), EGF-488 (Molecular Probes, cat. no. E13345, 1:300)) at 4 °C for 30 min. After washing with 1 ml of blocking buffer, labelled samples were centrifuged (300 xg, 10 min) and resuspended in 0.5 ml of blocking buffer for analysis. DAPI (0.1 µg/ml) was added to exclude dead cells from the analysis.

For proliferation analyses, immunostained cells were fixed with 100 µl of Cytofix/CytopermTM solution (BD Bioscience, cat. no. 554722) for 20 min at 4 °C. The fixative was washed with 3 ml of blocking solution and then samples were developed using 100 µl of the Click-iTTM Plus EdU Alexa FluorTM 555 Flow Cytometry Assay Kit (ThermoFisher, cat. no. C10638) following manufacturer's instructions.

For pNSC and aNSC sorting, cells were incubated with Myelin Removal beads (Miltenyi, cat. no. 130-096-731) passed through a previously equilibrated MS column (Miltenyi, cat. no. 130-042-401) on a MidiMACSTM magnetic separator (Miltenyi) following manufacturer's guidelines. Then, eluted fractions were collected, pelleted (300 xg, 10 min) and processed for fluorescent labeling as described above.

Instrument

LSR-Fortessa cytometer (Becton Dickinson) with 350, 405, 488, 561 and 640 nm lasers.
pNSC and aNSC cell fractions were isolated in a BD FACSAria III (350, 405, 488, 561 and 640 nm lasers) using a 100 µm nozzle at 20 psi.

Software

Data was collected with FACSDiva and analyzed with FlowJo 11.

Cell population abundance

The classifying strategy yields percentages of each population of interest relative to all live cells (selected by FSC, SSC and DAPI staining) that range approximately as follows: non-neurogenic astrocytes (2%–3%), NPC1 (2%–3%), NPC2 (2%–4%), NB1 (10%–12%), NB2 (40%–50%), and NSCs (10%–14%) distributed in qNSCs (4.5%–9%), pNSCs (1.8%–2.2%) and aNSCs (3%–4%).

Gating strategy

SEZ populations analysis and gating strategy is thoroughly detailed in Belenguer et al, Cell Stem Cell 28(2):285-299.e9, 2021; Belenguer et al, STAR Protoc 2(2):100425, 2021. Single SEZ cells were gated initially by size (FSC) and complexity (SSC) based on previous experiments excluding cell aggregates by SSC-A and SSC-H discordance and then using DAPI as a marker of non-viable cells. The following negative and positive gatings were stablished using a non-stained SEZ sample, the correspondent single stained controls and the Fluorescence Minus One (FMO) controls. First dead cells and some non-relevant cells are excluded by DAPI and staining of non-neurogenic cells using well stablished markers (CD45, CD31, Ter119, O4) and then, within the selected Lin- fraction, GLAST+ CD9high CD24- EGFR+/- corresponded to NSC, GLAST- EGFR+ CD24- or GLAST+ EGFR + CD24+ were selected as NPC, and GLAST- CD24+ EGFR+/- corresponded to NB (see Belenguer et al., Cell Stem Cell 28(2):285-299.e9, 2021 and Belenguer et al., STAR Protoc 2(2):100425, 2021).

☒ Tick this box to confirm that a figure exemplifying the gating strategy is provided in the Supplementary Information.
